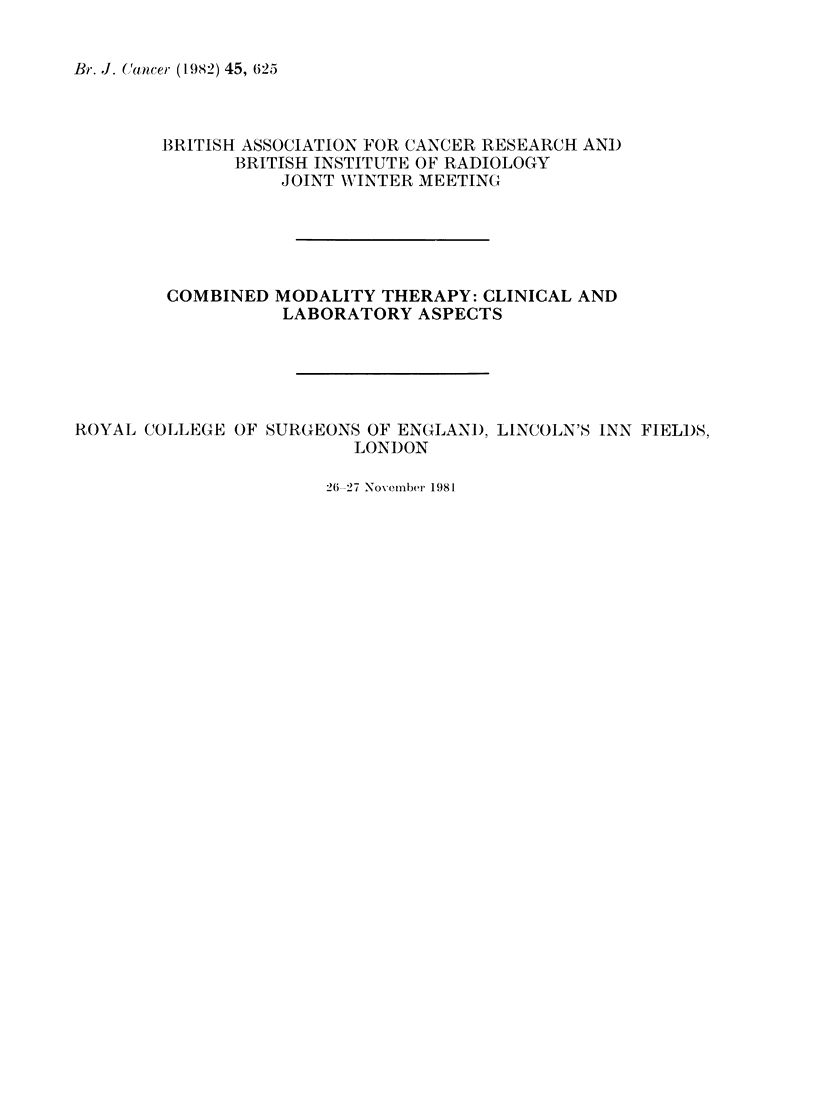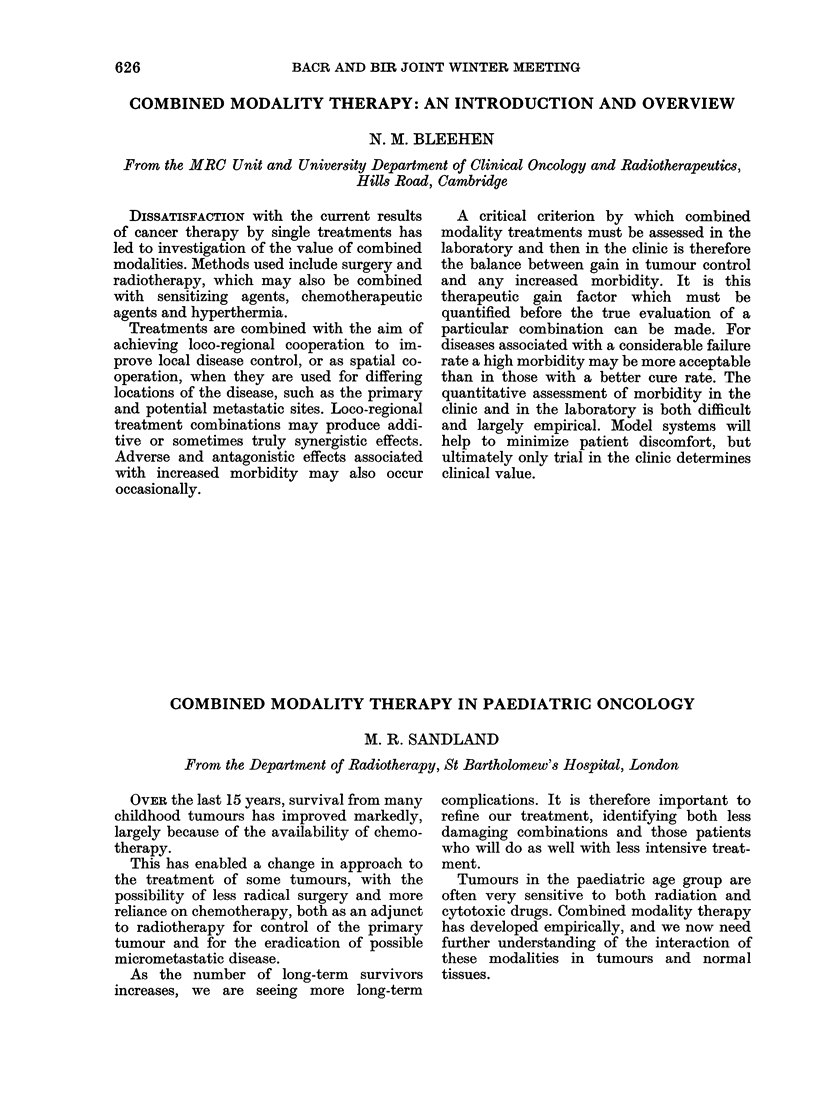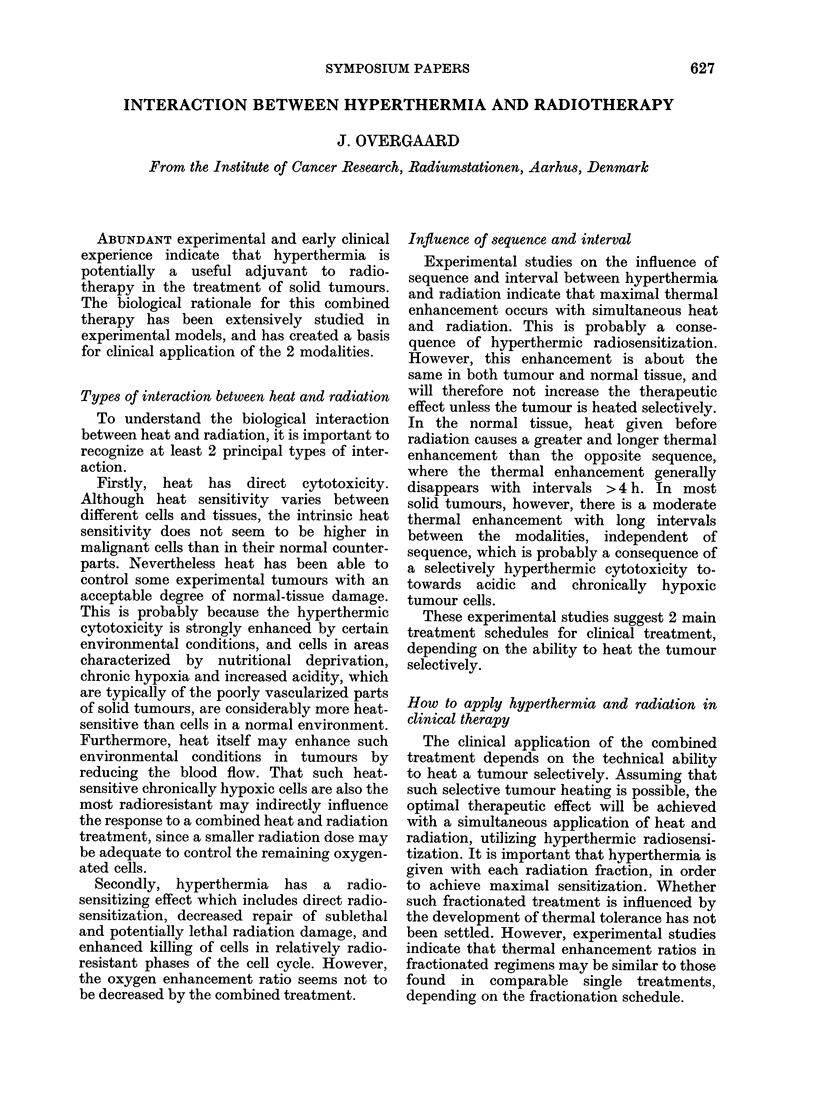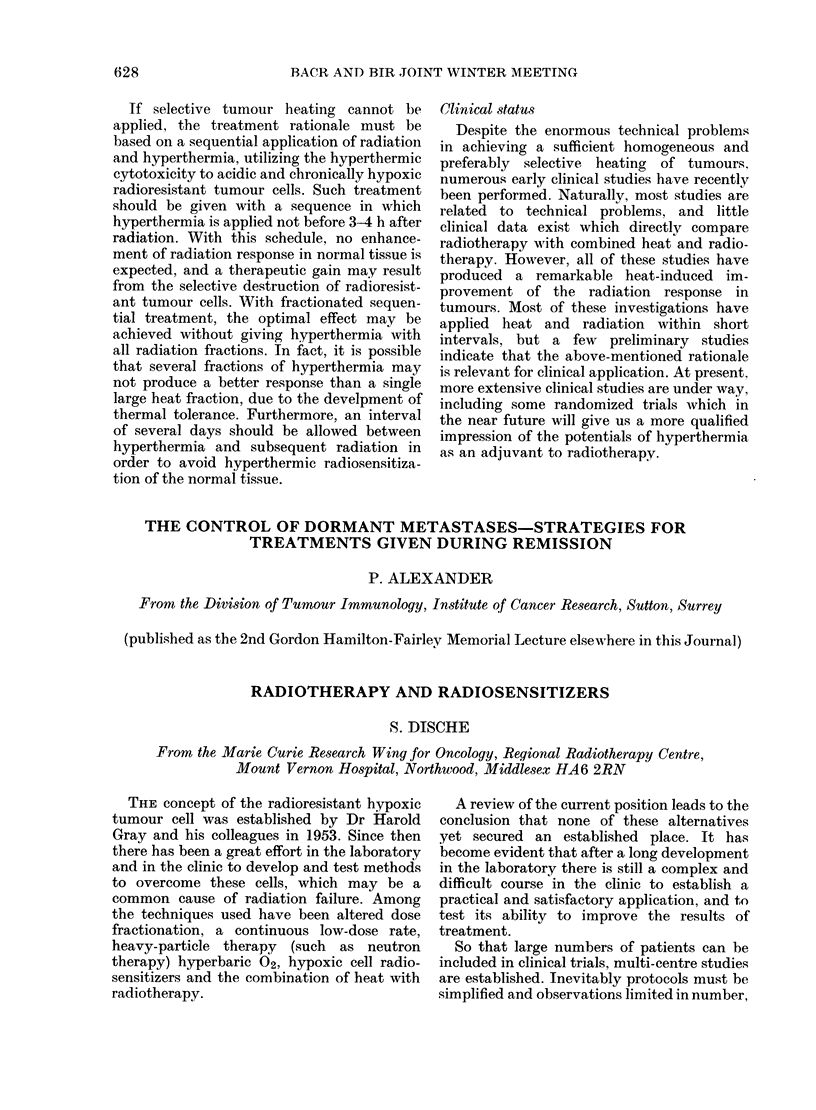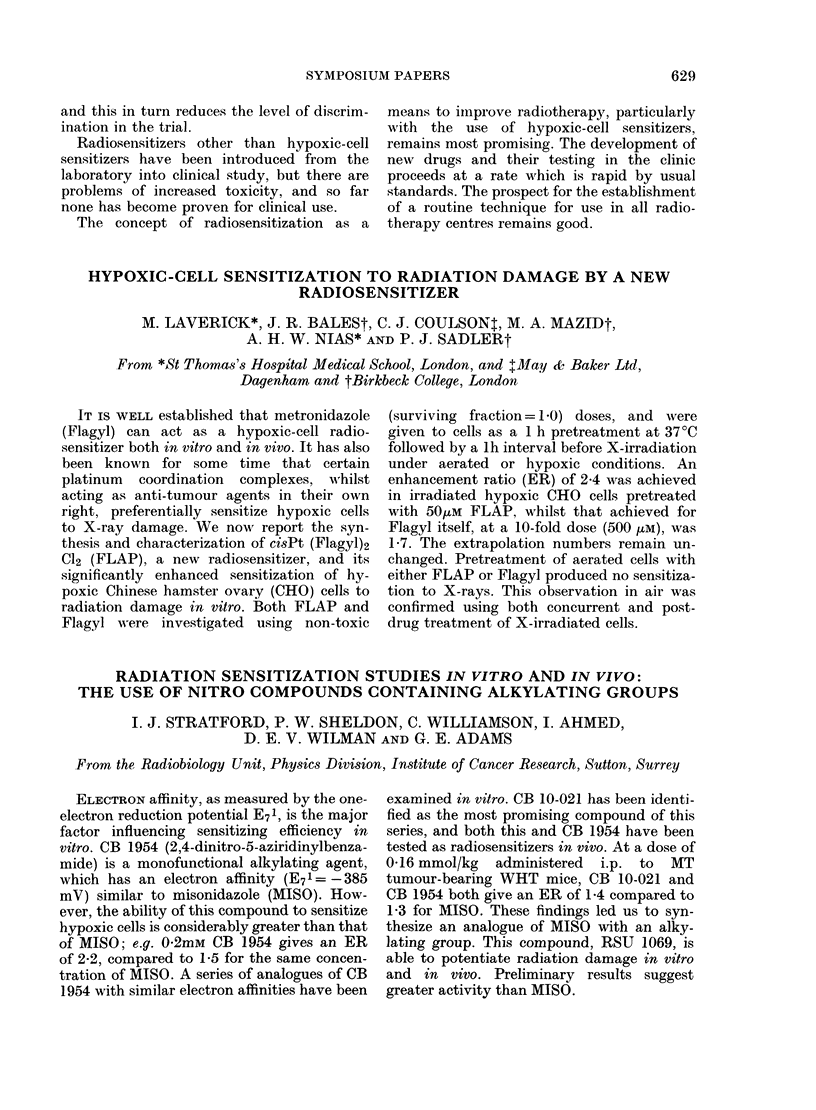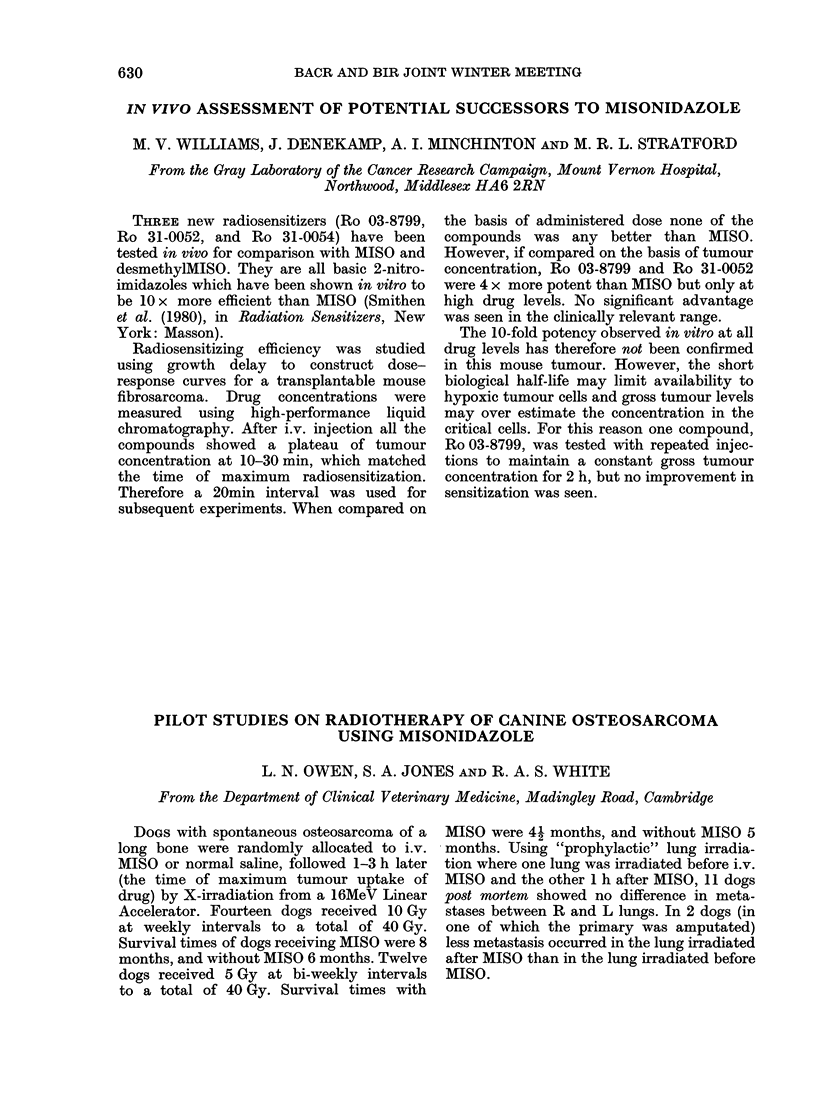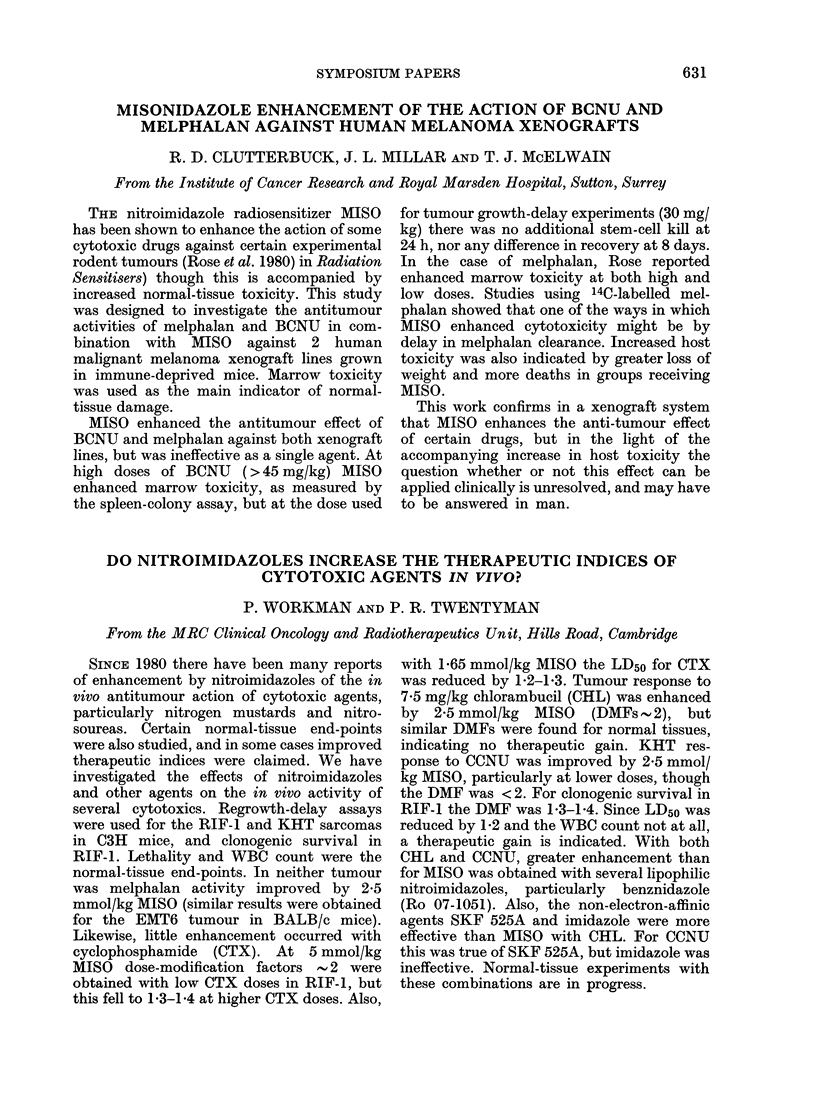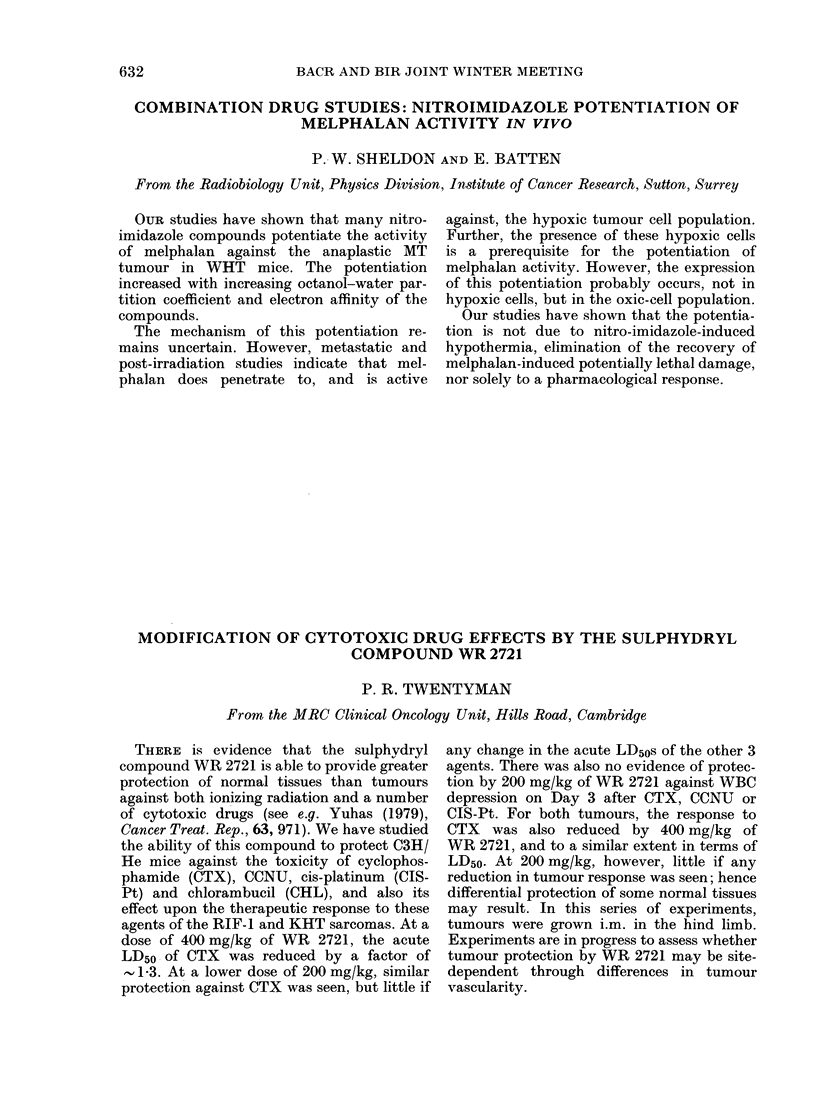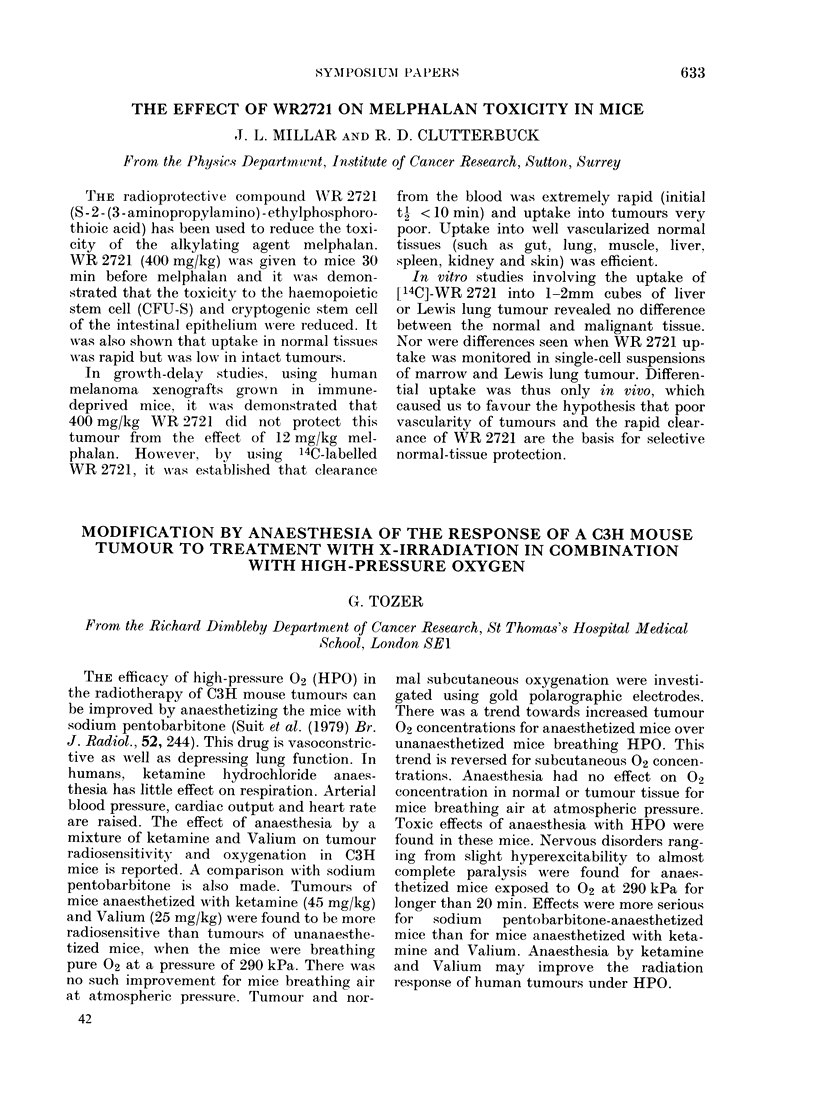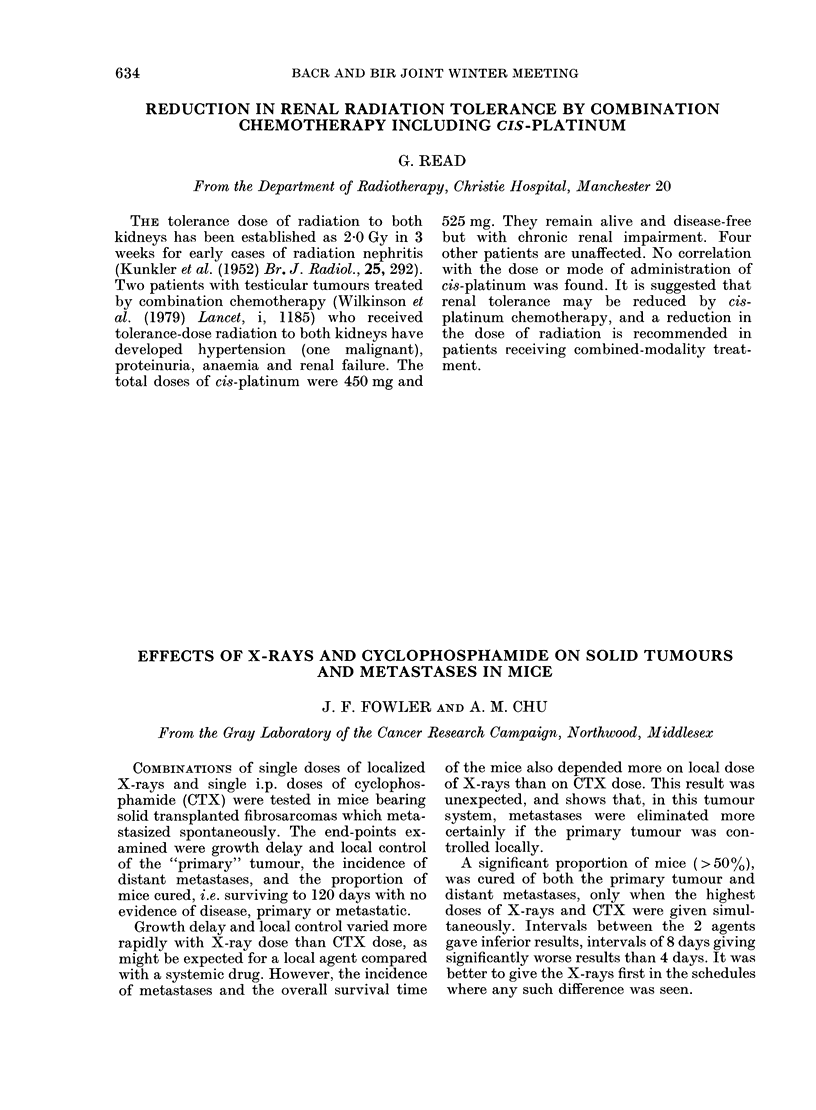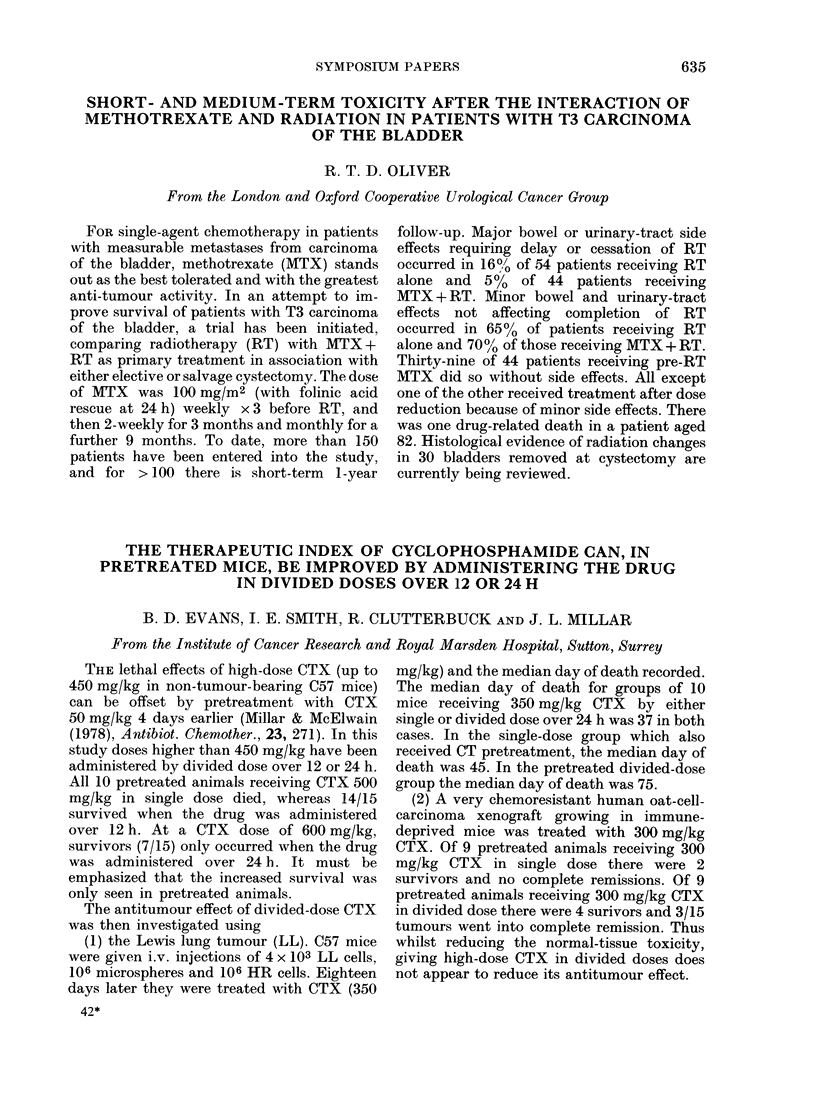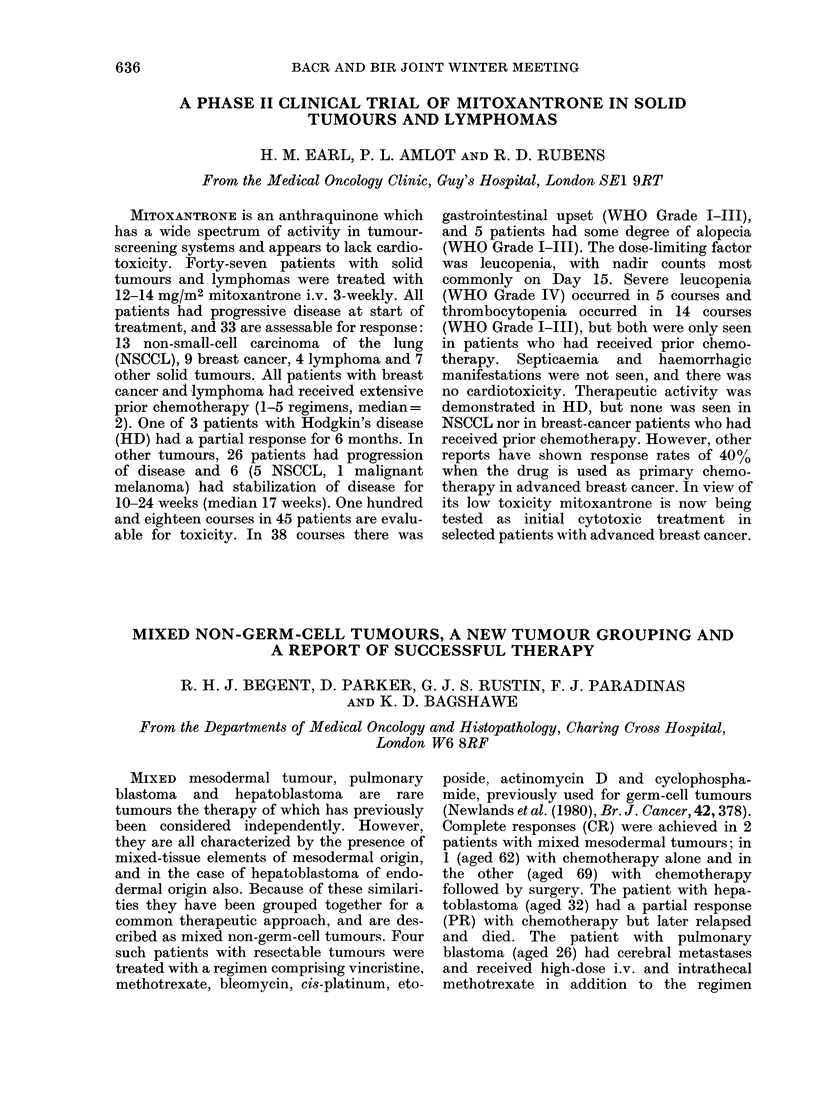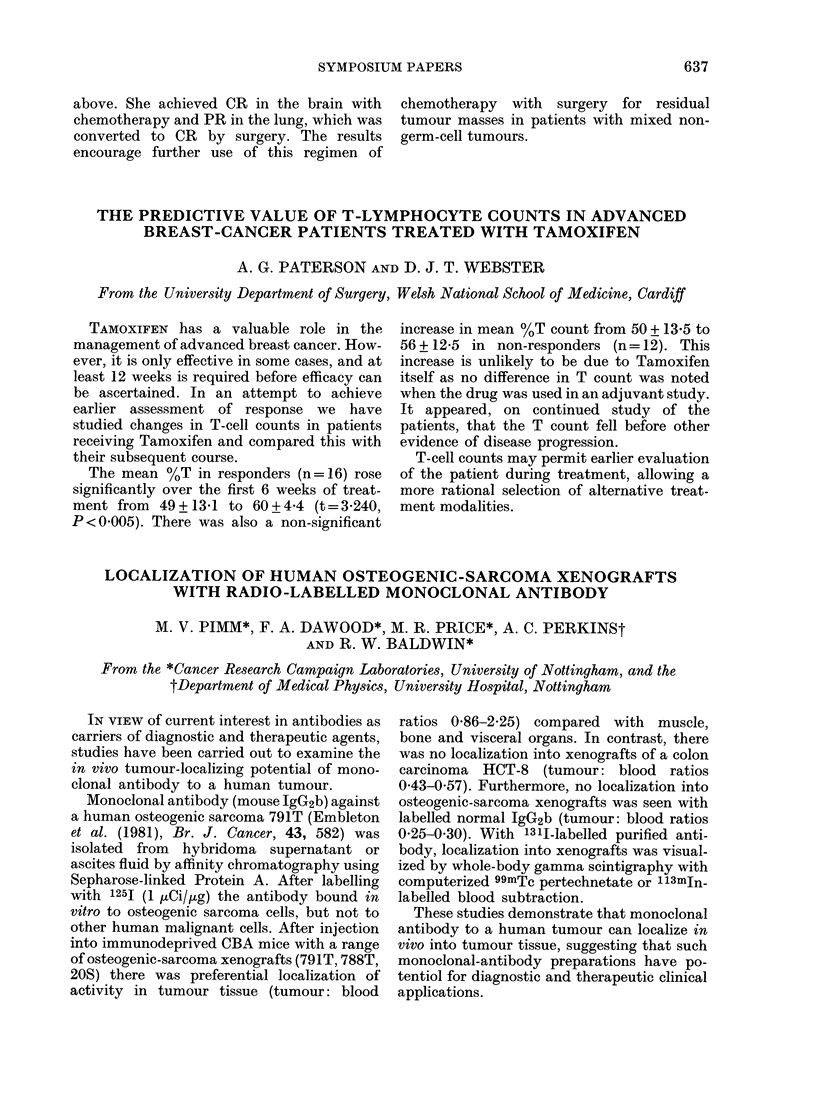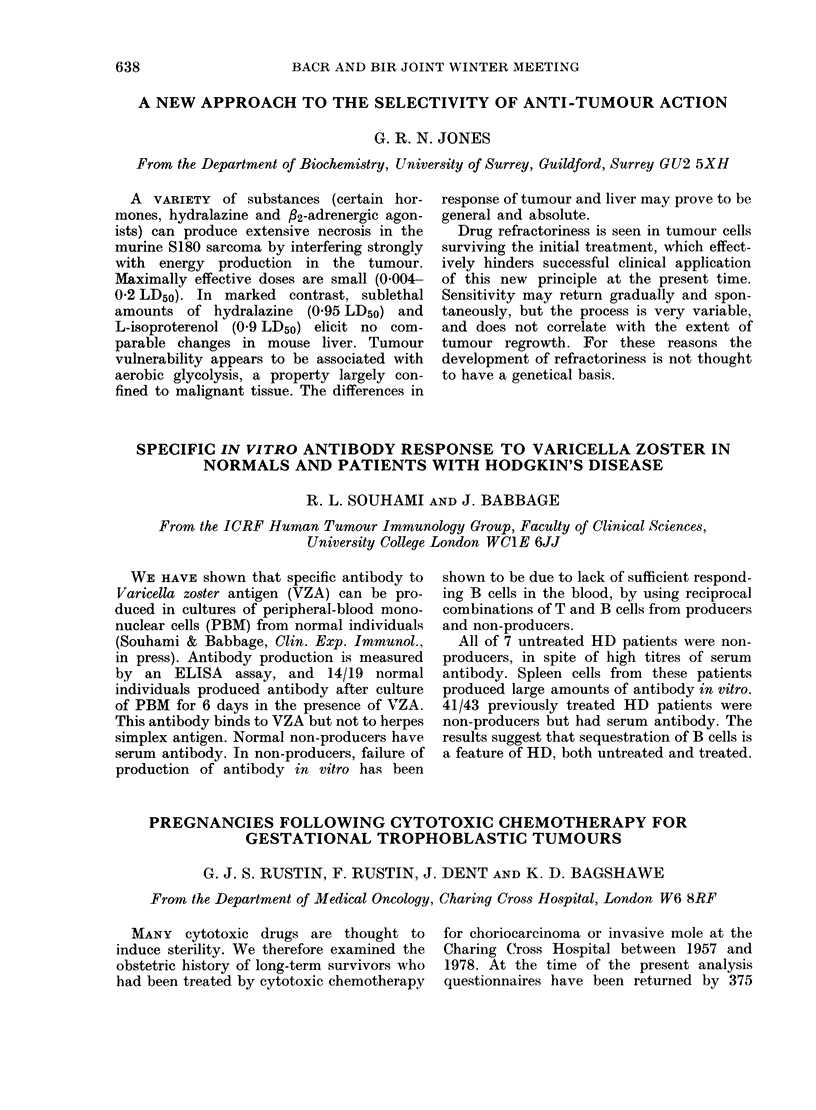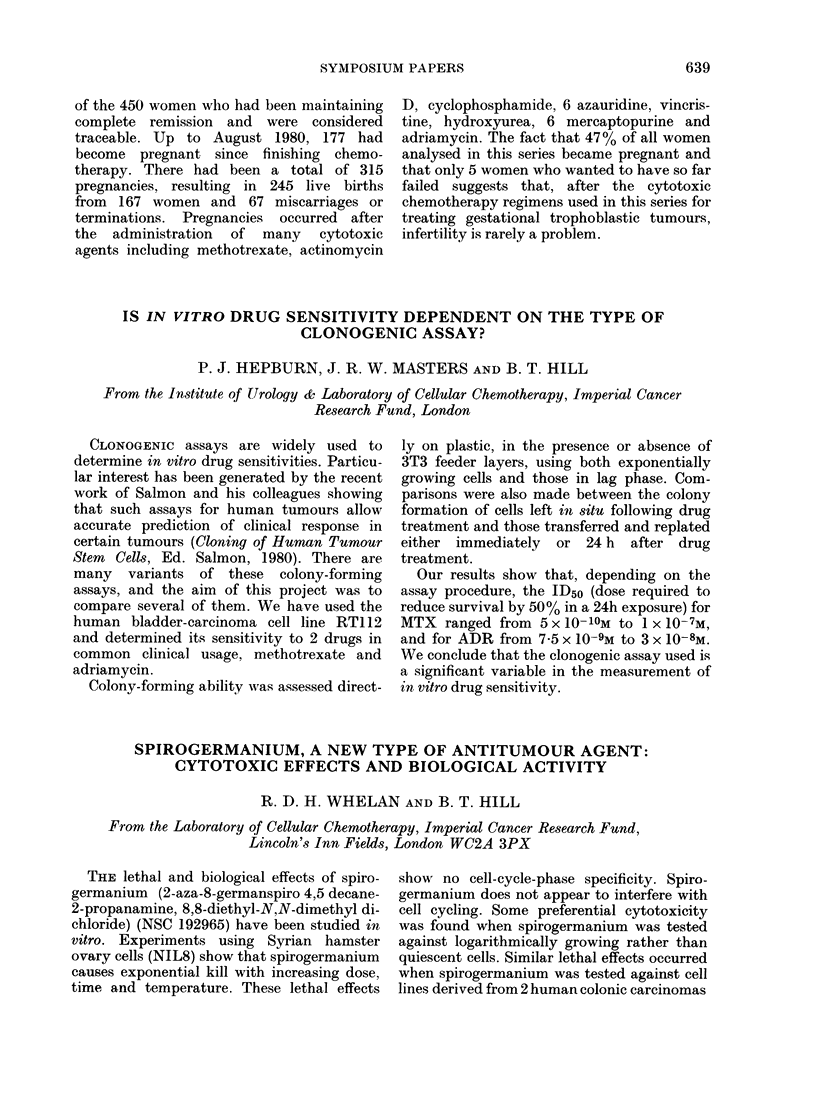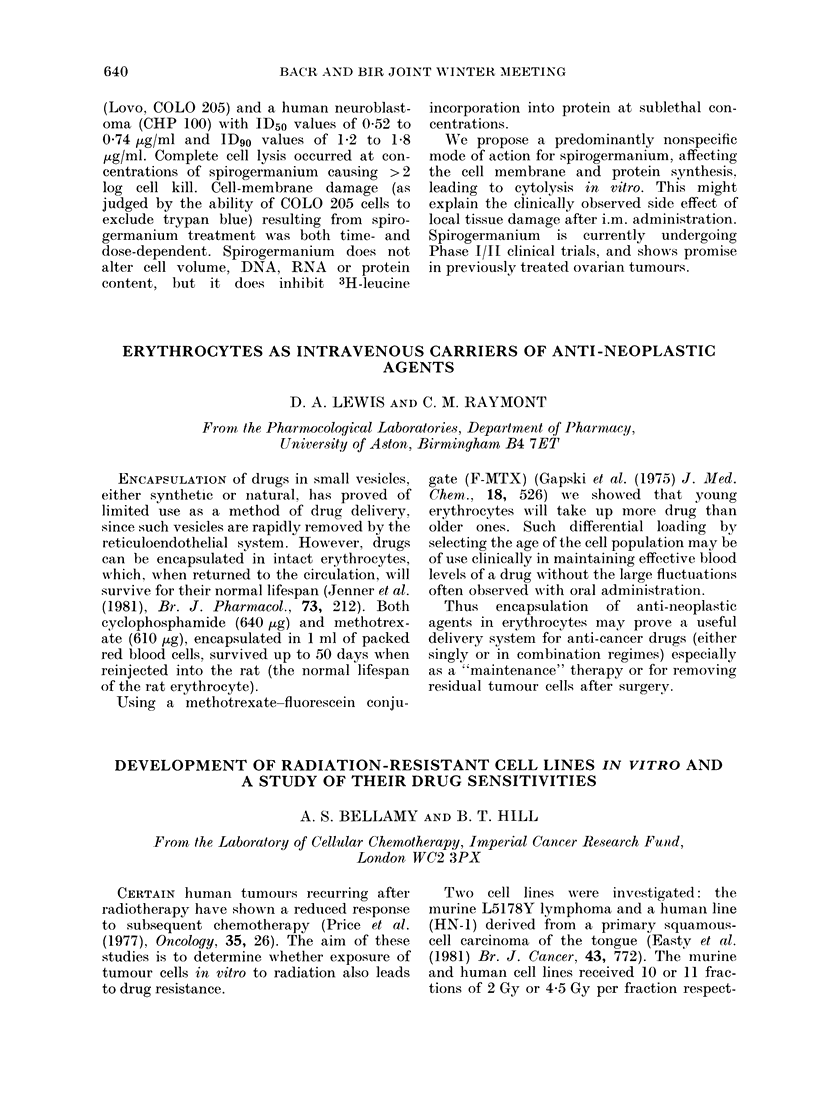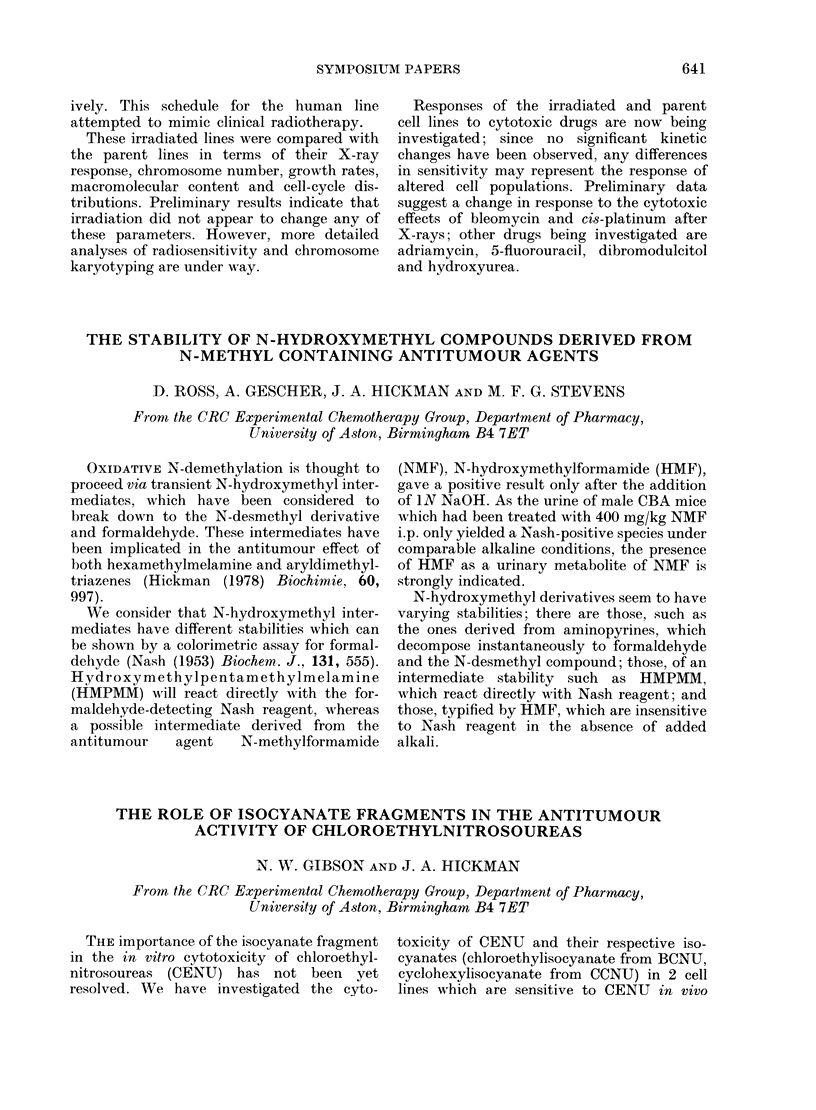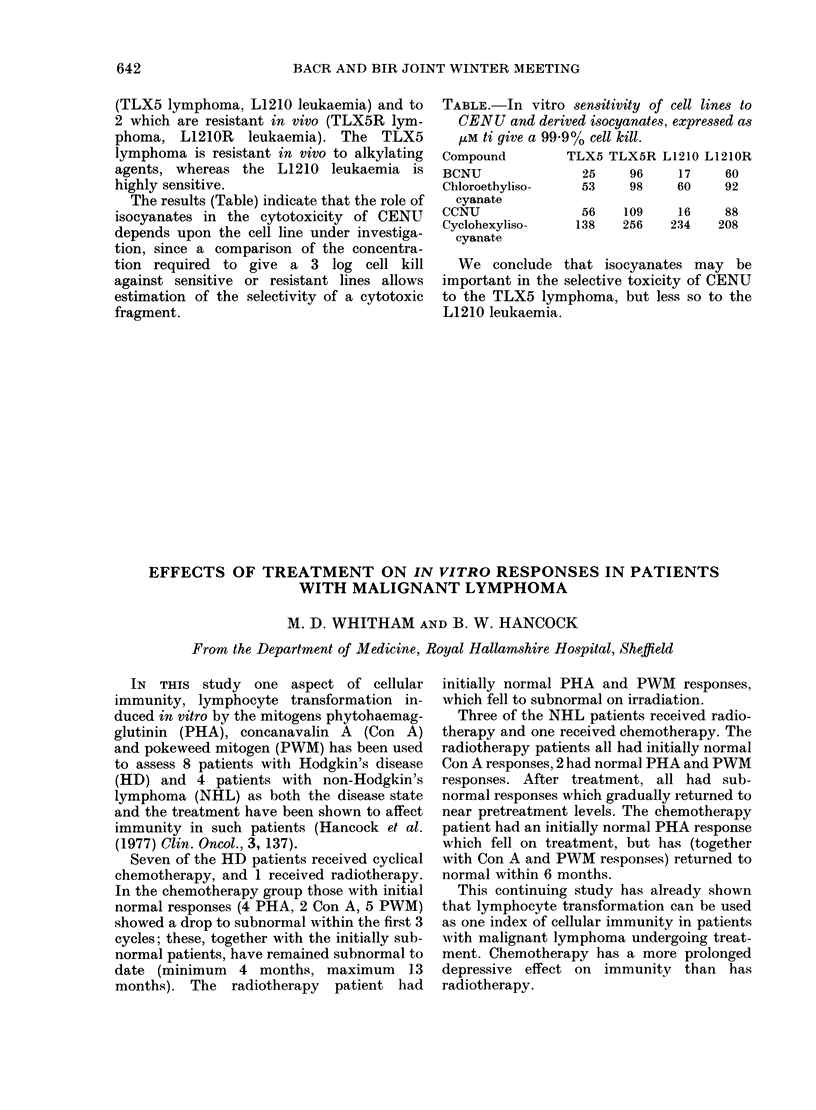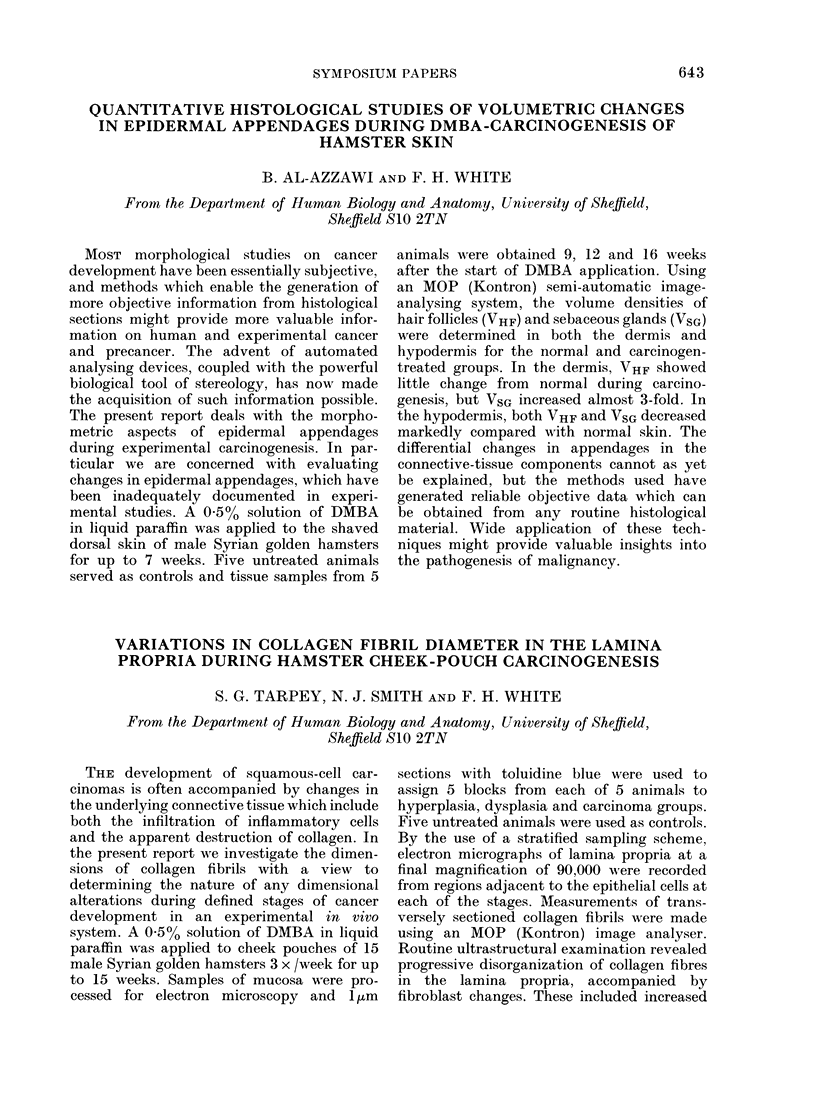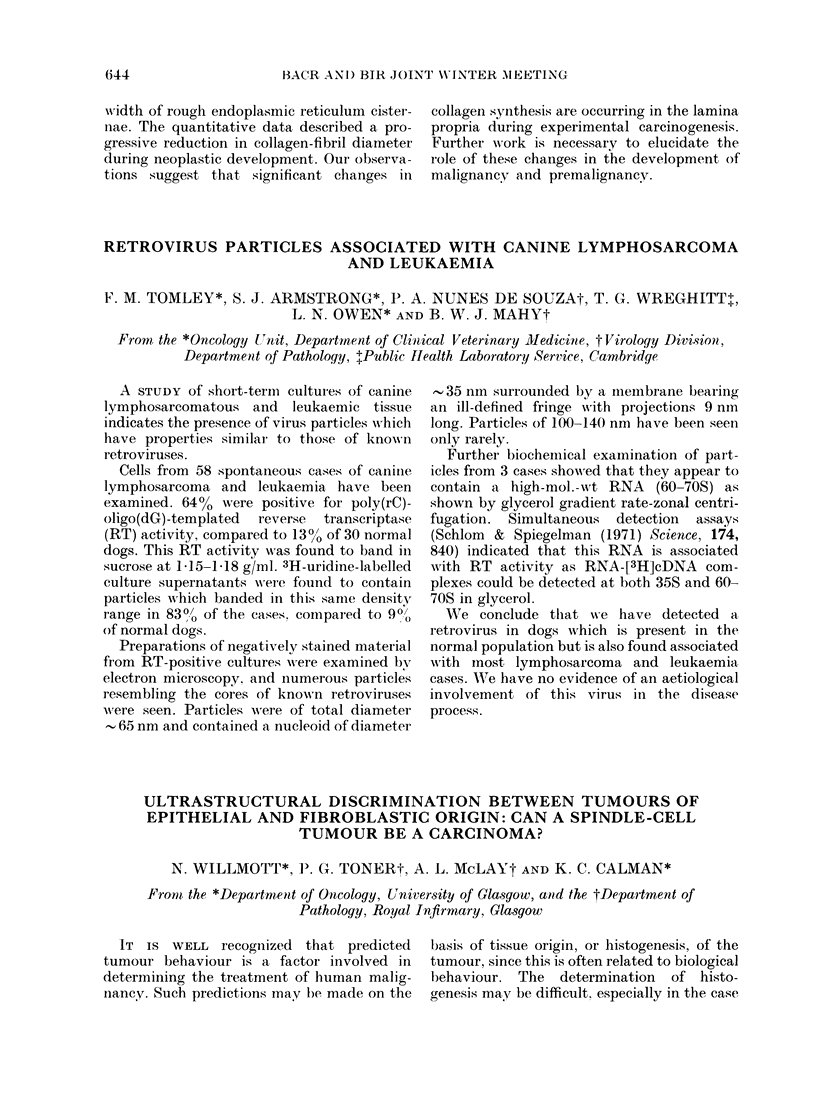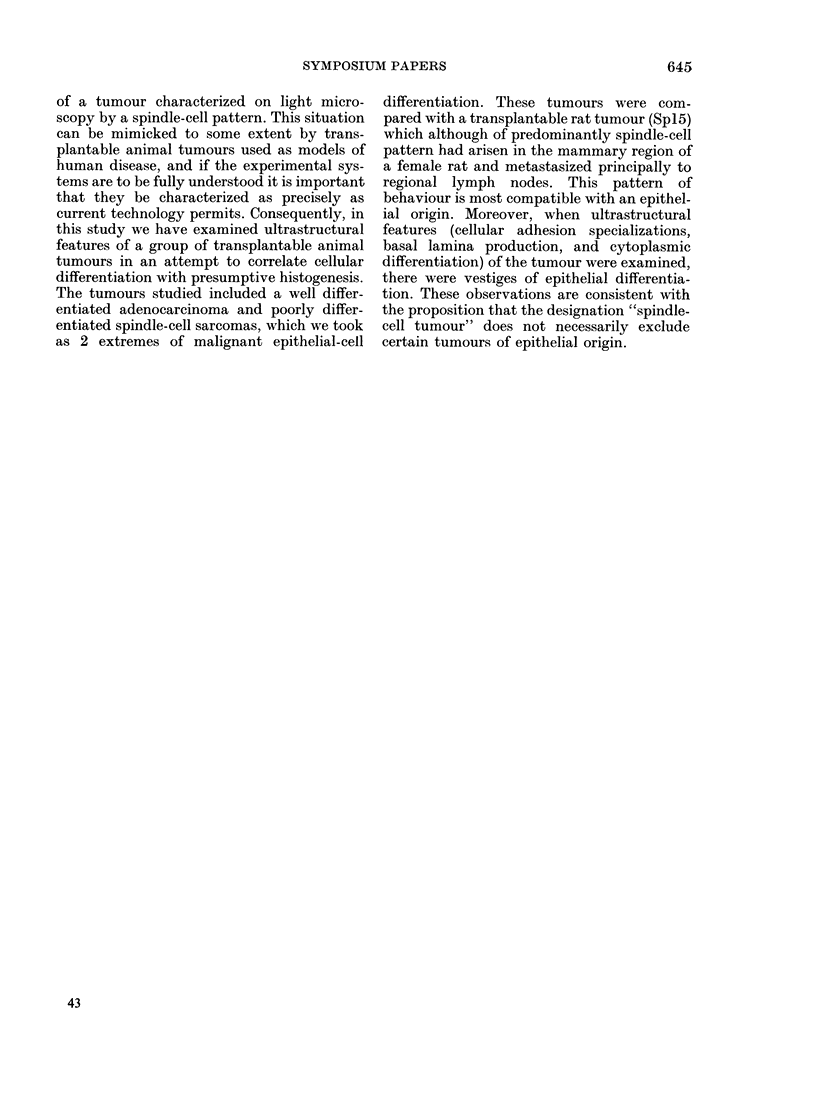# Combined modality therapy: clinical and laboratory aspects.

**DOI:** 10.1038/bjc.1982.102

**Published:** 1982-04

**Authors:** 


*Br. J. Cancer* (1982) **45**, 625

BRITISH ASSOCIATION FOR CANCER RESEARCH AND
BRITISH INSTITUTE OF RADIOLOGY
JOINT WINTER MEETING

---

### COMBINED MODALITY THERAPY: CLINICAL AND LABORATORY ASPECTS

---

ROYAL COLLEGE OF SURGEONS OF ENGLAND, LINCOLN'S INN FIELDS, LONDON

26–27 November 1981

### COMBINED MODALITY THERAPY: AN INTRODUCTION AND OVERVIEW

N. M. BLEEHEN

*From the MRC Unit and University Department of Clinical Oncology and Radiotherapeutics, Hills Road, Cambridge*

DISSATISFACTION with the current results of cancer therapy by single treatments has led to investigation of the value of combined modalities. Methods used include surgery and radiotherapy, which may also be combined with sensitizing agents, chemotherapeutic agents and hyperthermia.

Treatments are combined with the aim of achieving loco-regional cooperation to improve local disease control, or as spatial cooperation, when they are used for differing locations of the disease, such as the primary and potential metastatic sites. Loco-regional treatment combinations may produce additive or sometimes truly synergistic effects. Adverse and antagonistic effects associated with increased morbidity may also occur occasionally.

A critical criterion by which combined modality treatments must be assessed in the laboratory and then in the clinic is therefore the balance between gain in tumour control and any increased morbidity. It is this therapeutic gain factor which must be quantified before the true evaluation of a particular combination can be made. For diseases associated with a considerable failure rate a high morbidity may be more acceptable than in those with a better cure rate. The quantitative assessment of morbidity in the clinic and in the laboratory is both difficult and largely empirical. Model systems will help to minimize patient discomfort, but ultimately only trial in the clinic determines clinical value.

### COMBINED MODALITY THERAPY IN PAEDIATRIC ONCOLOGY

M. R. SANDLAND

*From the Department of Radiotherapy, St Bartholomew's Hospital, London*

OVER the last 15 years, survival from many childhood tumours has improved markedly, largely because of the availability of chemotherapy.

This has enabled a change in approach to the treatment of some tumours, with the possibility of less radical surgery and more reliance on chemotherapy, both as an adjunct to radiotherapy for control of the primary tumour and for the eradication of possible micrometastatic disease.

As the number of long-term survivors increases, we are seeing more long-term complications. It is therefore important to refine our treatment, identifying both less damaging combinations and those patients who will do as well with less intensive treatment.

Tumours in the paediatric age group are often very sensitive to both radiation and cytotoxic drugs. Combined modality therapy has developed empirically, and we now need further understanding of the interaction of these modalities in tumours and normal tissues.

### INTERACTION BETWEEN HYPERTHERMIA AND RADIOTHERAPY

J. OVERGAARD

*From the Institute of Cancer Research, Radiumstationen, Aarhus, Denmark*

ABUNDANT experimental and early clinical experience indicate that hyperthermia is potentially a useful adjuvant to radiotherapy in the treatment of solid tumours. The biological rationale for this combined therapy has been extensively studied in experimental models, and has created a basis for clinical application of the 2 modalities.

*Types of interaction between heat and radiation*

To understand the biological interaction between heat and radiation, it is important to recognize at least 2 principal types of interaction.

Firstly, heat has direct cytotoxicity. Although heat sensitivity varies between different cells and tissues, the intrinsic heat sensitivity does not seem to be higher in malignant cells than in their normal counterparts. Nevertheless heat has been able to control some experimental tumours with an acceptable degree of normal-tissue damage. This is probably because the hyperthermic cytotoxicity is strongly enhanced by certain environmental conditions, and cells in areas characterized by nutritional deprivation, chronic hypoxia and increased acidity, which are typically of the poorly vascularized parts of solid tumours, are considerably more heat-sensitive than cells in a normal environment. Furthermore, heat itself may enhance such environmental conditions in tumours by reducing the blood flow. That such heat-sensitive chronically hypoxic cells are also the most radioresistant may indirectly influence the response to a combined heat and radiation treatment, since a smaller radiation dose may be adequate to control the remaining oxygenated cells.

Secondly, hyperthermia has a radiosensitizing effect which includes direct radiosensitization, decreased repair of sublethal and potentially lethal radiation damage, and enhanced killing of cells in relatively radioresistant phases of the cell cycle. However, the oxygen enhancement ratio seems not to be decreased by the combined treatment.

*Influence of sequence and interval*

Experimental studies on the influence of sequence and interval between hyperthermia and radiation indicate that maximal thermal enhancement occurs with simultaneous heat and radiation. This is probably a consequence of hyperthermic radiosensitization. However, this enhancement is about the same in both tumour and normal tissue, and will therefore not increase the therapeutic effect unless the tumour is heated selectively. In the normal tissue, heat given before radiation causes a greater and longer thermal enhancement than the opposite sequence, where the thermal enhancement generally disappears with intervals >4 h. In most solid tumours, however, there is a moderate thermal enhancement with long intervals between the modalities, independent of sequence, which is probably a consequence of a selectively hyperthermic cytotoxicity to-towards acidic and chronically hypoxic tumour cells.

These experimental studies suggest 2 main treatment schedules for clinical treatment, depending on the ability to heat the tumour selectively.

*How to apply hyperthermia and radiation in clinical therapy*

The clinical application of the combined treatment depends on the technical ability to heat a tumour selectively. Assuming that such selective tumour heating is possible, the optimal therapeutic effect will be achieved with a simultaneous application of heat and radiation, utilizing hyperthermic radiosensitization. It is important that hyperthermia is given with each radiation fraction, in order to achieve maximal sensitization. Whether such fractionated treatment is influenced by the development of thermal tolerance has not been settled. However, experimental studies indicate that thermal enhancement ratios in fractionated regimens may be similar to those found in comparable single treatments, depending on the fractionation schedule.

If selective tumour heating cannot be applied, the treatment rationale must be based on a sequential application of radiation and hyperthermia, utilizing the hyperthermic cytotoxicity to acidic and chronically hypoxic radioresistant tumour cells. Such treatment should be given with a sequence in which hyperthermia is applied not before 3–4 h after radiation. With this schedule, no enhancement of radiation response in normal tissue is expected, and a therapeutic gain may result from the selective destruction of radioresistant tumour cells. With fractionated sequential treatment, the optimal effect may be achieved without giving hyperthermia with all radiation fractions. In fact, it is possible that several fractions of hyperthermia may not produce a better response than a single large heat fraction, due to the develpment of thermal tolerance. Furthermore, an interval of several days should be allowed between hyperthermia and subsequent radiation in order to avoid hyperthermic radiosensitization of the normal tissue.

*Clinical status*

Despite the enormous technical problems in achieving a sufficient homogeneous and preferably selective heating of tumours, numerous early clinical studies have recently been performed. Naturally, most studies are related to technical problems, and little clinical data exist which directly compare radiotherapy with combined heat and radiotherapy. However, all of these studies have produced a remarkable heat-induced improvement of the radiation response in tumours. Most of these investigations have applied heat and radiation within short intervals, but a few preliminary studies indicate that the above-mentioned rationale is relevant for clinical application. At present, more extensive clinical studies are under way, including some randomized trials which in the near future will give us a more qualified impression of the potentials of hyperthermia as an adjuvant to radiotherapy.

### THE CONTROL OF DORMANT METASTASES—STRATEGIES FOR TREATMENTS GIVEN DURING REMISSION

P. ALEXANDER

*From the Division of Tumour Immunology, Institute of Cancer Research, Sutton, Surrey*

(published as the 2nd Gordon Hamilton-Fairley Memorial Lecture elsewhere in this Journal)

### RADIOTHERAPY AND RADIOSENSITIZERS

S. DISCHE

*From the Marie Curie Research Wing for Oncology, Regional Radiotherapy Centre, Mount Vernon Hospital, Northwood, Middlesex HA6 2RN*

THE concept of the radioresistant hypoxic tumour cell was established by Dr Harold Gray and his colleagues in 1953. Since then there has been a great effort in the laboratory and in the clinic to develop and test methods to overcome these cells, which may be a common cause of radiation failure. Among the techniques used have been altered dose fractionation, a continuous low-dose rate, heavy-particle therapy (such as neutron therapy) hyperbaric $O_2$, hypoxic cell radiosensitizers and the combination of heat with radiotherapy.

A review of the current position leads to the conclusion that none of these alternatives yet secured an established place. It has become evident that after a long development in the laboratory there is still a complex and difficult course in the clinic to establish a practical and satisfactory application, and to test its ability to improve the results of treatment.

So that large numbers of patients can be included in clinical trials, multi-centre studies are established. Inevitably protocols must be simplified and observations limited in number, and this in turn reduces the level of discrimination in the trial.

Radiosensitizers other than hypoxic-cell sensitizers have been introduced from the laboratory into clinical study, but there are problems of increased toxicity, and so far none has become proven for clinical use.

The concept of radiosensitization as a means to improve radiotherapy, particularly with the use of hypoxic-cell sensitizers, remains most promising. The development of new drugs and their testing in the clinic proceeds at a rate which is rapid by usual standards. The prospect for the establishment of a routine technique for use in all radiotherapy centres remains good.

### HYPOXIC-CELL SENSITIZATION TO RADIATION DAMAGE BY A NEW RADIOSENSITIZER

M. LAVERICK*, J. R. BALES†, C. J. COULSON‡, M. A. MAZID†, A. H. W. NIAS* AND P. J. SADLER†

*From *St Thomas's Hospital Medical School, London, and ‡May & Baker Ltd, Dagenham and †Birkbeck College, London*

It is well established that metronidazole (Flagyl) can act as a hypoxic-cell radiosensitizer both *in vitro* and *in vivo*. It has also been known for some time that certain platinum coordination complexes, whilst acting as anti-tumour agents in their own right, preferentially sensitize hypoxic cells to X-ray damage. We now report the synthesis and characterization of *cis*Pt $(Flagyl)_2$ $Cl_2$ (FLAP), a new radiosensitizer, and its significantly enhanced sensitization of hypoxic Chinese hamster ovary (CHO) cells to radiation damage *in vitro*. Both FLAP and Flagyl were investigated using non-toxic (surviving fraction = 1·0) doses, and were given to cells as a 1 h pretreatment at 37°C followed by a 1h interval before X-irradiation under aerated or hypoxic conditions. An enhancement ratio (ER) of 2·4 was achieved in irradiated hypoxic CHO cells pretreated with 50$\mu$M FLAP, whilst that achieved for Flagyl itself, at a 10-fold dose (500 $\mu$M), was 1·7. The extrapolation numbers remain unchanged. Pretreatment of aerated cells with either FLAP or Flagyl produced no sensitization to X-rays. This observation in air was confirmed using both concurrent and post-drug treatment of X-irradiated cells.

### RADIATION SENSITIZATION STUDIES *IN VITRO* AND *IN VIVO*: THE USE OF NITRO COMPOUNDS CONTAINING ALKYLATING GROUPS

I. J. STRATFORD, P. W. SHELDON, C. WILLIAMSON, I. AHMED, D. E. V. WILMAN AND G. E. ADAMS

*From the Radiobiology Unit, Physics Division, Institute of Cancer Research, Sutton, Surrey*

Electron affinity, as measured by the one-electron reduction potential $E_7{}^1$, is the major factor influencing sensitizing efficiency *in vitro*. CB 1954 (2,4-dinitro-5-aziridinylbenzamide) is a monofunctional alkylating agent, which has an electron affinity ($E_7{}^1 = -385$ mV) similar to misonidazole (MISO). However, the ability of this compound to sensitize hypoxic cells is considerably greater than that of MISO; *e.g.* 0·2mM CB 1954 gives an ER of 2·2, compared to 1·5 for the same concentration of MISO. A series of analogues of CB 1954 with similar electron affinities have been examined *in vitro*. CB 10-021 has been identified as the most promising compound of this series, and both this and CB 1954 have been tested as radiosensitizers *in vivo*. At a dose of 0·16 mmol/kg administered i.p. to MT tumour-bearing WHT mice, CB 10-021 and CB 1954 both give an ER of 1·4 compared to 1·3 for MISO. These findings led us to synthesize an analogue of MISO with an alkylating group. This compound, RSU 1069, is able to potentiate radiation damage *in vitro* and *in vivo*. Preliminary results suggest greater activity than MISO.

### *IN VIVO* ASSESSMENT OF POTENTIAL SUCCESSORS TO MISONIDAZOLE

M. V. WILLIAMS, J. DENEKAMP, A. I. MINCHINTON AND M. R. L. STRATFORD

*From the Gray Laboratory of the Cancer Research Campaign, Mount Vernon Hospital, Northwood, Middlesex HA6 2RN*

THREE new radiosensitizers (Ro 03-8799, Ro 31-0052, and Ro 31-0054) have been tested *in vivo* for comparison with MISO and desmethylMISO. They are all basic 2-nitroimidazoles which have been shown *in vitro* to be 10× more efficient than MISO (Smithen *et al.* (1980), in *Radiation Sensitizers*, New York: Masson).

Radiosensitizing efficiency was studied using growth delay to construct dose–response curves for a transplantable mouse fibrosarcoma. Drug concentrations were measured using high-performance liquid chromatography. After i.v. injection all the compounds showed a plateau of tumour concentration at 10–30 min, which matched the time of maximum radiosensitization. Therefore a 20min interval was used for subsequent experiments. When compared on the basis of administered dose none of the compounds was any better than MISO. However, if compared on the basis of tumour concentration, Ro 03-8799 and Ro 31-0052 were 4× more potent than MISO but only at high drug levels. No significant advantage was seen in the clinically relevant range.

The 10-fold potency observed *in vitro* at all drug levels has therefore *not* been confirmed in this mouse tumour. However, the short biological half-life may limit availability to hypoxic tumour cells and gross tumour levels may over estimate the concentration in the critical cells. For this reason one compound, Ro 03-8799, was tested with repeated injections to maintain a constant gross tumour concentration for 2 h, but no improvement in sensitization was seen.

### PILOT STUDIES ON RADIOTHERAPY OF CANINE OSTEOSARCOMA USING MISONIDAZOLE

L. N. OWEN, S. A. JONES AND R. A. S. WHITE

*From the Department of Clinical Veterinary Medicine, Madingley Road, Cambridge*

DOGS with spontaneous osteosarcoma of a long bone were randomly allocated to i.v. MISO or normal saline, followed 1–3 h later (the time of maximum tumour uptake of drug) by X-irradiation from a 16MeV Linear Accelerator. Fourteen dogs received 10 Gy at weekly intervals to a total of 40 Gy. Survival times of dogs receiving MISO were 8 months, and without MISO 6 months. Twelve dogs received 5 Gy at bi-weekly intervals to a total of 40 Gy. Survival times with MISO were 4½ months, and without MISO 5 months. Using "prophylactic" lung irradiation where one lung was irradiated before i.v. MISO and the other 1 h after MISO, 11 dogs *post mortem* showed no difference in metastases between R and L lungs. In 2 dogs (in one of which the primary was amputated) less metastasis occurred in the lung irradiated after MISO than in the lung irradiated before MISO.

### MISONIDAZOLE ENHANCEMENT OF THE ACTION OF BCNU AND MELPHALAN AGAINST HUMAN MELANOMA XENOGRAFTS

R. D. CLUTTERBUCK, J. L. MILLAR AND T. J. McELWAIN

*From the Institute of Cancer Research and Royal Marsden Hospital, Sutton, Surrey*

THE nitroimidazole radiosensitizer MISO has been shown to enhance the action of some cytotoxic drugs against certain experimental rodent tumours (Rose *et al.* 1980) in *Radiation Sensitisers*) though this is accompanied by increased normal-tissue toxicity. This study was designed to investigate the antitumour activities of melphalan and BCNU in combination with MISO against 2 human malignant melanoma xenograft lines grown in immune-deprived mice. Marrow toxicity was used as the main indicator of normal-tissue damage.

MISO enhanced the antitumour effect of BCNU and melphalan against both xenograft lines, but was ineffective as a single agent. At high doses of BCNU ($>45$ mg/kg) MISO enhanced marrow toxicity, as measured by the spleen-colony assay, but at the dose used for tumour growth-delay experiments (30 mg/kg) there was no additional stem-cell kill at 24 h, nor any difference in recovery at 8 days. In the case of melphalan, Rose reported enhanced marrow toxicity at both high and low doses. Studies using $^{14}C$-labelled melphalan showed that one of the ways in which MISO enhanced cytotoxicity might be by delay in melphalan clearance. Increased host toxicity was also indicated by greater loss of weight and more deaths in groups receiving MISO.

This work confirms in a xenograft system that MISO enhances the anti-tumour effect of certain drugs, but in the light of the accompanying increase in host toxicity the question whether or not this effect can be applied clinically is unresolved, and may have to be answered in man.

### DO NITROIMIDAZOLES INCREASE THE THERAPEUTIC INDICES OF CYTOTOXIC AGENTS *IN VIVO*?

P. WORKMAN AND P. R. TWENTYMAN

*From the MRC Clinical Oncology and Radiotherapeutics Unit, Hills Road, Cambridge*

SINCE 1980 there have been many reports of enhancement by nitroimidazoles of the *in vivo* antitumour action of cytotoxic agents, particularly nitrogen mustards and nitrosoureas. Certain normal-tissue end-points were also studied, and in some cases improved therapeutic indices were claimed. We have investigated the effects of nitroimidazoles and other agents on the *in vivo* activity of several cytotoxics. Regrowth-delay assays were used for the RIF-1 and KHT sarcomas in C3H mice, and clonogenic survival in RIF-1. Lethality and WBC count were the normal-tissue end-points. In neither tumour was melphalan activity improved by 2·5 mmol/kg MISO (similar results were obtained for the EMT6 tumour in BALB/c mice). Likewise, little enhancement occurred with cyclophosphamide (CTX). At 5 mmol/kg MISO dose-modification factors $\sim 2$ were obtained with low CTX doses in RIF-1, but this fell to 1·3–1·4 at higher CTX doses. Also, with 1·65 mmol/kg MISO the $LD_{50}$ for CTX was reduced by 1·2–1·3. Tumour response to 7·5 mg/kg chlorambucil (CHL) was enhanced by 2·5 mmol/kg MISO (DMFs $\sim 2$), but similar DMFs were found for normal tissues, indicating no therapeutic gain. KHT response to CCNU was improved by 2·5 mmol/kg MISO, particularly at lower doses, though the DMF was $<2$. For clonogenic survival in RIF-1 the DMF was 1·3–1·4. Since $LD_{50}$ was reduced by 1·2 and the WBC count not at all, a therapeutic gain is indicated. With both CHL and CCNU, greater enhancement than for MISO was obtained with several lipophilic nitroimidazoles, particularly benznidazole (Ro 07-1051). Also, the non-electron-affinic agents SKF 525A and imidazole were more effective than MISO with CHL. For CCNU this was true of SKF 525A, but imidazole was ineffective. Normal-tissue experiments with these combinations are in progress.

### COMBINATION DRUG STUDIES: NITROIMIDAZOLE POTENTIATION OF MELPHALAN ACTIVITY *IN VIVO*

P. W. SHELDON AND E. BATTEN

*From the Radiobiology Unit, Physics Division, Institute of Cancer Research, Sutton, Surrey*

OUR studies have shown that many nitroimidazole compounds potentiate the activity of melphalan against the anaplastic MT tumour in WHT mice. The potentiation increased with increasing octanol–water partition coefficient and electron affinity of the compounds.

The mechanism of this potentiation remains uncertain. However, metastatic and post-irradiation studies indicate that melphalan does penetrate to, and is active against, the hypoxic tumour cell population. Further, the presence of these hypoxic cells is a prerequisite for the potentiation of melphalan activity. However, the expression of this potentiation probably occurs, not in hypoxic cells, but in the oxic-cell population.

Our studies have shown that the potentiation is not due to nitro-imidazole-induced hypothermia, elimination of the recovery of melphalan-induced potentially lethal damage, nor solely to a pharmacological response.

### MODIFICATION OF CYTOTOXIC DRUG EFFECTS BY THE SULPHYDRYL COMPOUND WR 2721

P. R. TWENTYMAN

*From the MRC Clinical Oncology Unit, Hills Road, Cambridge*

THERE is evidence that the sulphydryl compound WR 2721 is able to provide greater protection of normal tissues than tumours against both ionizing radiation and a number of cytotoxic drugs (see *e.g.* Yuhas (1979), *Cancer Treat. Rep.*, **63,** 971). We have studied the ability of this compound to protect C3H/He mice against the toxicity of cyclophosphamide (CTX), CCNU, cis-platinum (CIS-Pt) and chlorambucil (CHL), and also its effect upon the therapeutic response to these agents of the RIF-1 and KHT sarcomas. At a dose of 400 mg/kg of WR 2721, the acute $LD_{50}$ of CTX was reduced by a factor of ~1·3. At a lower dose of 200 mg/kg, similar protection against CTX was seen, but little if any change in the acute $LD_{50}$s of the other 3 agents. There was also no evidence of protection by 200 mg/kg of WR 2721 against WBC depression on Day 3 after CTX, CCNU or CIS-Pt. For both tumours, the response to CTX was also reduced by 400 mg/kg of WR 2721, and to a similar extent in terms of $LD_{50}$. At 200 mg/kg, however, little if any reduction in tumour response was seen; hence differential protection of some normal tissues may result. In this series of experiments, tumours were grown i.m. in the hind limb. Experiments are in progress to assess whether tumour protection by WR 2721 may be site-dependent through differences in tumour vascularity.

### THE EFFECT OF WR2721 ON MELPHALAN TOXICITY IN MICE

J. L. MILLAR AND R. D. CLUTTERBUCK

*From the Physics Departmwnt, Institute of Cancer Research, Sutton, Surrey*

THE radioprotective compound WR 2721 (S-2-(3-aminopropylamino)-ethylphosphorothioic acid) has been used to reduce the toxicity of the alkylating agent melphalan. WR 2721 (400 mg/kg) was given to mice 30 min before melphalan and it was demonstrated that the toxicity to the haemopoietic stem cell (CFU-S) and cryptogenic stem cell of the intestinal epithelium were reduced. It was also shown that uptake in normal tissues was rapid but was low in intact tumours.

In growth-delay studies, using human melanoma xenografts grown in immune-deprived mice, it was demonstrated that 400 mg/kg WR 2721 did not protect this tumour from the effect of 12 mg/kg melphalan. However, by using $^{14}C$-labelled WR 2721, it was established that clearance from the blood was extremely rapid (initial $t_{\frac{1}{2}} < 10$ min) and uptake into tumours very poor. Uptake into well vascularized normal tissues (such as gut, lung, muscle, liver, spleen, kidney and skin) was efficient.

*In vitro* studies involving the uptake of [$^{14}C$]-WR 2721 into 1–2mm cubes of liver or Lewis lung tumour revealed no difference between the normal and malignant tissue. Nor were differences seen when WR 2721 uptake was monitored in single-cell suspensions of marrow and Lewis lung tumour. Differential uptake was thus only *in vivo*, which caused us to favour the hypothesis that poor vascularity of tumours and the rapid clearance of WR 2721 are the basis for selective normal-tissue protection.

### MODIFICATION BY ANAESTHESIA OF THE RESPONSE OF A C3H MOUSE TUMOUR TO TREATMENT WITH X-IRRADIATION IN COMBINATION WITH HIGH-PRESSURE OXYGEN

G. TOZER

*From the Richard Dimbleby Department of Cancer Research, St Thomas's Hospital Medical School, London SE1*

THE efficacy of high-pressure $O_2$ (HPO) in the radiotherapy of C3H mouse tumours can be improved by anaesthetizing the mice with sodium pentobarbitone (Suit *et al.* (1979) *Br. J. Radiol.*, **52**, 244). This drug is vasoconstrictive as well as depressing lung function. In humans, ketamine hydrochloride anaesthesia has little effect on respiration. Arterial blood pressure, cardiac output and heart rate are raised. The effect of anaesthesia by a mixture of ketamine and Valium on tumour radiosensitivity and oxygenation in C3H mice is reported. A comparison with sodium pentobarbitone is also made. Tumours of mice anaesthetized with ketamine (45 mg/kg) and Valium (25 mg/kg) were found to be more radiosensitive than tumours of unanaesthetized mice, when the mice were breathing pure $O_2$ at a pressure of 290 kPa. There was no such improvement for mice breathing air at atmospheric pressure. Tumour and normal subcutaneous oxygenation were investigated using gold polarographic electrodes. There was a trend towards increased tumour $O_2$ concentrations for anaesthetized mice over unanaesthetized mice breathing HPO. This trend is reversed for subcutaneous $O_2$ concentrations. Anaesthesia had no effect on $O_2$ concentration in normal or tumour tissue for mice breathing air at atmospheric pressure. Toxic effects of anaesthesia with HPO were found in these mice. Nervous disorders ranging from slight hyperexcitability to almost complete paralysis were found for anaesthetized mice exposed to $O_2$ at 290 kPa for longer than 20 min. Effects were more serious for sodium pentobarbitone-anaesthetized mice than for mice anaesthetized with ketamine and Valium. Anaesthesia by ketamine and Valium may improve the radiation response of human tumours under HPO.

### REDUCTION IN RENAL RADIATION TOLERANCE BY COMBINATION CHEMOTHERAPY INCLUDING *CIS*-PLATINUM

G. READ

*From the Department of Radiotherapy, Christie Hospital, Manchester* 20

THE tolerance dose of radiation to both kidneys has been established as 2·0 Gy in 3 weeks for early cases of radiation nephritis (Kunkler *et al.* (1952) *Br. J. Radiol.*, **25**, 292). Two patients with testicular tumours treated by combination chemotherapy (Wilkinson *et al.* (1979) *Lancet*, i, 1185) who received tolerance-dose radiation to both kidneys have developed hypertension (one malignant), proteinuria, anaemia and renal failure. The total doses of *cis*-platinum were 450 mg and 525 mg. They remain alive and disease-free but with chronic renal impairment. Four other patients are unaffected. No correlation with the dose or mode of administration of *cis*-platinum was found. It is suggested that renal tolerance may be reduced by *cis*-platinum chemotherapy, and a reduction in the dose of radiation is recommended in patients receiving combined-modality treatment.

### EFFECTS OF X-RAYS AND CYCLOPHOSPHAMIDE ON SOLID TUMOURS AND METASTASES IN MICE

J. F. FOWLER AND A. M. CHU

*From the Gray Laboratory of the Cancer Research Campaign, Northwood, Middlesex*

COMBINATIONS of single doses of localized X-rays and single i.p. doses of cyclophosphamide (CTX) were tested in mice bearing solid transplanted fibrosarcomas which metastasized spontaneously. The end-points examined were growth delay and local control of the "primary" tumour, the incidence of distant metastases, and the proportion of mice cured, *i.e.* surviving to 120 days with no evidence of disease, primary or metastatic.

Growth delay and local control varied more rapidly with X-ray dose than CTX dose, as might be expected for a local agent compared with a systemic drug. However, the incidence of metastases and the overall survival time of the mice also depended more on local dose of X-rays than on CTX dose. This result was unexpected, and shows that, in this tumour system, metastases were eliminated more certainly if the primary tumour was controlled locally.

A significant proportion of mice (>50%), was cured of both the primary tumour and distant metastases, only when the highest doses of X-rays and CTX were given simultaneously. Intervals between the 2 agents gave inferior results, intervals of 8 days giving significantly worse results than 4 days. It was better to give the X-rays first in the schedules where any such difference was seen.

### SHORT- AND MEDIUM-TERM TOXICITY AFTER THE INTERACTION OF METHOTREXATE AND RADIATION IN PATIENTS WITH T3 CARCINOMA OF THE BLADDER

R. T. D. OLIVER

*From the London and Oxford Cooperative Urological Cancer Group*

FOR single-agent chemotherapy in patients with measurable metastases from carcinoma of the bladder, methotrexate (MTX) stands out as the best tolerated and with the greatest anti-tumour activity. In an attempt to improve survival of patients with T3 carcinoma of the bladder, a trial has been initiated, comparing radiotherapy (RT) with MTX+RT as primary treatment in association with either elective or salvage cystectomy. The dose of MTX was 100 mg/m$^2$ (with folinic acid rescue at 24 h) weekly $\times 3$ before RT, and then 2-weekly for 3 months and monthly for a further 9 months. To date, more than 150 patients have been entered into the study, and for >100 there is short-term 1-year follow-up. Major bowel or urinary-tract side effects requiring delay or cessation of RT occurred in 16% of 54 patients receiving RT alone and 5% of 44 patients receiving MTX+RT. Minor bowel and urinary-tract effects not affecting completion of RT occurred in 65% of patients receiving RT alone and 70% of those receiving MTX+RT. Thirty-nine of 44 patients receiving pre-RT MTX did so without side effects. All except one of the other received treatment after dose reduction because of minor side effects. There was one drug-related death in a patient aged 82. Histological evidence of radiation changes in 30 bladders removed at cystectomy are currently being reviewed.

### THE THERAPEUTIC INDEX OF CYCLOPHOSPHAMIDE CAN, IN PRETREATED MICE, BE IMPROVED BY ADMINISTERING THE DRUG IN DIVIDED DOSES OVER 12 OR 24 H

B. D. EVANS, I. E. SMITH, R. CLUTTERBUCK AND J. L. MILLAR

*From the Institute of Cancer Research and Royal Marsden Hospital, Sutton, Surrey*

THE lethal effects of high-dose CTX (up to 450 mg/kg in non-tumour-bearing C57 mice) can be offset by pretreatment with CTX 50 mg/kg 4 days earlier (Millar & McElwain (1978), *Antibiot. Chemother.*, **23,** 271). In this study doses higher than 450 mg/kg have been administered by divided dose over 12 or 24 h. All 10 pretreated animals receiving CTX 500 mg/kg in single dose died, whereas 14/15 survived when the drug was administered over 12 h. At a CTX dose of 600 mg/kg, survivors (7/15) only occurred when the drug was administered over 24 h. It must be emphasized that the increased survival was only seen in pretreated animals.

The antitumour effect of divided-dose CTX was then investigated using

(1) the Lewis lung tumour (LL). C57 mice were given i.v. injections of $4 \times 10^3$ LL cells, $10^6$ microspheres and $10^6$ HR cells. Eighteen days later they were treated with CTX (350 mg/kg) and the median day of death recorded. The median day of death for groups of 10 mice receiving 350 mg/kg CTX by either single or divided dose over 24 h was 37 in both cases. In the single-dose group which also received CT pretreatment, the median day of death was 45. In the pretreated divided-dose group the median day of death was 75.

(2) A very chemoresistant human oat-cell-carcinoma xenograft growing in immune-deprived mice was treated with 300 mg/kg CTX. Of 9 pretreated animals receiving 300 mg/kg CTX in single dose there were 2 survivors and no complete remissions. Of 9 pretreated animals receiving 300 mg/kg CTX in divided dose there were 4 surivors and 3/15 tumours went into complete remission. Thus whilst reducing the normal-tissue toxicity, giving high-dose CTX in divided doses does not appear to reduce its antitumour effect.

### A PHASE II CLINICAL TRIAL OF MITOXANTRONE IN SOLID TUMOURS AND LYMPHOMAS

H. M. EARL, P. L. AMLOT AND R. D. RUBENS

*From the Medical Oncology Clinic, Guy's Hospital, London SE1 9RT*

MITOXANTRONE is an anthraquinone which has a wide spectrum of activity in tumour-screening systems and appears to lack cardiotoxicity. Forty-seven patients with solid tumours and lymphomas were treated with 12–14 mg/m$^2$ mitoxantrone i.v. 3-weekly. All patients had progressive disease at start of treatment, and 33 are assessable for response: 13 non-small-cell carcinoma of the lung (NSCCL), 9 breast cancer, 4 lymphoma and 7 other solid tumours. All patients with breast cancer and lymphoma had received extensive prior chemotherapy (1–5 regimens, median = 2). One of 3 patients with Hodgkin's disease (HD) had a partial response for 6 months. In other tumours, 26 patients had progression of disease and 6 (5 NSCCL, 1 malignant melanoma) had stabilization of disease for 10–24 weeks (median 17 weeks). One hundred and eighteen courses in 45 patients are evaluable for toxicity. In 38 courses there was gastrointestinal upset (WHO Grade I–III), and 5 patients had some degree of alopecia (WHO Grade I–III). The dose-limiting factor was leucopenia, with nadir counts most commonly on Day 15. Severe leucopenia (WHO Grade IV) occurred in 5 courses and thrombocytopenia occurred in 14 courses (WHO Grade I–III), but both were only seen in patients who had received prior chemotherapy. Septicaemia and haemorrhagic manifestations were not seen, and there was no cardiotoxicity. Therapeutic activity was demonstrated in HD, but none was seen in NSCCL nor in breast-cancer patients who had received prior chemotherapy. However, other reports have shown response rates of 40% when the drug is used as primary chemotherapy in advanced breast cancer. In view of its low toxicity mitoxantrone is now being tested as initial cytotoxic treatment in selected patients with advanced breast cancer.

### MIXED NON-GERM-CELL TUMOURS, A NEW TUMOUR GROUPING AND A REPORT OF SUCCESSFUL THERAPY

R. H. J. BEGENT, D. PARKER, G. J. S. RUSTIN, F. J. PARADINAS AND K. D. BAGSHAWE

*From the Departments of Medical Oncology and Histopathology, Charing Cross Hospital, London W6 8RF*

MIXED mesodermal tumour, pulmonary blastoma and hepatoblastoma are rare tumours the therapy of which has previously been considered independently. However, they are all characterized by the presence of mixed-tissue elements of mesodermal origin, and in the case of hepatoblastoma of endodermal origin also. Because of these similarities they have been grouped together for a common therapeutic approach, and are described as mixed non-germ-cell tumours. Four such patients with resectable tumours were treated with a regimen comprising vincristine, methotrexate, bleomycin, *cis*-platinum, etoposide, actinomycin D and cyclophosphamide, previously used for germ-cell tumours (Newlands *et al.* (1980), *Br. J. Cancer*, **42**, 378). Complete responses (CR) were achieved in 2 patients with mixed mesodermal tumours; in 1 (aged 62) with chemotherapy alone and in the other (aged 69) with chemotherapy followed by surgery. The patient with hepatoblastoma (aged 32) had a partial response (PR) with chemotherapy but later relapsed and died. The patient with pulmonary blastoma (aged 26) had cerebral metastases and received high-dose i.v. and intrathecal methotrexate in addition to the regimen above. She achieved CR in the brain with chemotherapy and PR in the lung, which was converted to CR by surgery. The results encourage further use of this regimen of chemotherapy with surgery for residual tumour masses in patients with mixed non-germ-cell tumours.

### THE PREDICTIVE VALUE OF T-LYMPHOCYTE COUNTS IN ADVANCED BREAST-CANCER PATIENTS TREATED WITH TAMOXIFEN

A. G. PATERSON AND D. J. T. WEBSTER

*From the University Department of Surgery, Welsh National School of Medicine, Cardiff*

TAMOXIFEN has a valuable role in the management of advanced breast cancer. However, it is only effective in some cases, and at least 12 weeks is required before efficacy can be ascertained. In an attempt to achieve earlier assessment of response we have studied changes in T-cell counts in patients receiving Tamoxifen and compared this with their subsequent course.

The mean %T in responders (n = 16) rose significantly over the first 6 weeks of treatment from $49 \pm 13{\cdot}1$ to $60 \pm 4{\cdot}4$ ($t = 3{\cdot}240$, $P < 0{\cdot}005$). There was also a non-significant increase in mean %T count from $50 \pm 13{\cdot}5$ to $56 \pm 12{\cdot}5$ in non-responders (n = 12). This increase is unlikely to be due to Tamoxifen itself as no difference in T count was noted when the drug was used in an adjuvant study. It appeared, on continued study of the patients, that the T count fell before other evidence of disease progression.

T-cell counts may permit earlier evaluation of the patient during treatment, allowing a more rational selection of alternative treatment modalities.

### LOCALIZATION OF HUMAN OSTEOGENIC-SARCOMA XENOGRAFTS WITH RADIO-LABELLED MONOCLONAL ANTIBODY

M. V. PIMM*, F. A. DAWOOD*, M. R. PRICE*, A. C. PERKINS† AND R. W. BALDWIN*

*From the *Cancer Research Campaign Laboratories, University of Nottingham, and the †Department of Medical Physics, University Hospital, Nottingham*

IN VIEW of current interest in antibodies as carriers of diagnostic and therapeutic agents, studies have been carried out to examine the *in vivo* tumour-localizing potential of monoclonal antibody to a human tumour.

Monoclonal antibody (mouse $IgG_2b$) against a human osteogenic sarcoma 791T (Embleton *et al.* (1981), *Br. J. Cancer*, **43,** 582) was isolated from hybridoma supernatant or ascites fluid by affinity chromatography using Sepharose-linked Protein A. After labelling with $^{125}I$ (1 $\mu Ci/\mu g$) the antibody bound *in vitro* to osteogenic sarcoma cells, but not to other human malignant cells. After injection into immunodeprived CBA mice with a range of osteogenic-sarcoma xenografts (791T, 788T, 20S) there was preferential localization of activity in tumour tissue (tumour: blood ratios 0·86–2·25) compared with muscle, bone and visceral organs. In contrast, there was no localization into xenografts of a colon carcinoma HCT-8 (tumour: blood ratios 0·43–0·57). Furthermore, no localization into osteogenic-sarcoma xenografts was seen with labelled normal $IgG_2b$ (tumour: blood ratios 0·25–0·30). With $^{131}I$-labelled purified antibody, localization into xenografts was visualized by whole-body gamma scintigraphy with computerized $^{99m}Tc$ pertechnetate or $^{113m}In$-labelled blood subtraction.

These studies demonstrate that monoclonal antibody to a human tumour can localize *in vivo* into tumour tissue, suggesting that such monoclonal-antibody preparations have potentiol for diagnostic and therapeutic clinical applications.

### A NEW APPROACH TO THE SELECTIVITY OF ANTI-TUMOUR ACTION

G. R. N. JONES

*From the Department of Biochemistry, University of Surrey, Guildford, Surrey GU2 5XH*

A VARIETY of substances (certain hormones, hydralazine and $\beta_2$-adrenergic agonists) can produce extensive necrosis in the murine S180 sarcoma by interfering strongly with energy production in the tumour. Maximally effective doses are small (0·004–0·2 $LD_{50}$). In marked contrast, sublethal amounts of hydralazine (0·95 $LD_{50}$) and L-isoproterenol (0·9 $LD_{50}$) elicit no comparable changes in mouse liver. Tumour vulnerability appears to be associated with aerobic glycolysis, a property largely confined to malignant tissue. The differences in response of tumour and liver may prove to be general and absolute.

Drug refractoriness is seen in tumour cells surviving the initial treatment, which effectively hinders successful clinical application of this new principle at the present time. Sensitivity may return gradually and spontaneously, but the process is very variable, and does not correlate with the extent of tumour regrowth. For these reasons the development of refractoriness is not thought to have a genetical basis.

### SPECIFIC *IN VITRO* ANTIBODY RESPONSE TO VARICELLA ZOSTER IN NORMALS AND PATIENTS WITH HODGKIN'S DISEASE

R. L. SOUHAMI AND J. BABBAGE

*From the ICRF Human Tumour Immunology Group, Faculty of Clinical Sciences, University College London WC1E 6JJ*

WE HAVE shown that specific antibody to *Varicella zoster* antigen (VZA) can be produced in cultures of peripheral-blood mononuclear cells (PBM) from normal individuals (Souhami & Babbage, *Clin. Exp. Immunol.*, in press). Antibody production is measured by an ELISA assay, and 14/19 normal individuals produced antibody after culture of PBM for 6 days in the presence of VZA. This antibody binds to VZA but not to herpes simplex antigen. Normal non-producers have serum antibody. In non-producers, failure of production of antibody *in vitro* has been shown to be due to lack of sufficient responding B cells in the blood, by using reciprocal combinations of T and B cells from producers and non-producers.

All of 7 untreated HD patients were non-producers, in spite of high titres of serum antibody. Spleen cells from these patients produced large amounts of antibody *in vitro*. 41/43 previously treated HD patients were non-producers but had serum antibody. The results suggest that sequestration of B cells is a feature of HD, both untreated and treated.

### PREGNANCIES FOLLOWING CYTOTOXIC CHEMOTHERAPY FOR GESTATIONAL TROPHOBLASTIC TUMOURS

G. J. S. RUSTIN, F. RUSTIN, J. DENT AND K. D. BAGSHAWE

*From the Department of Medical Oncology, Charing Cross Hospital, London W6 8RF*

MANY cytotoxic drugs are thought to induce sterility. We therefore examined the obstetric history of long-term survivors who had been treated by cytotoxic chemotherapy for choriocarcinoma or invasive mole at the Charing Cross Hospital between 1957 and 1978. At the time of the present analysis questionnaires have been returned by 375

of the 450 women who had been maintaining complete remission and were considered traceable. Up to August 1980, 177 had become pregnant since finishing chemotherapy. There had been a total of 315 pregnancies, resulting in 245 live births from 167 women and 67 miscarriages or terminations. Pregnancies occurred after the administration of many cytotoxic agents including methotrexate, actinomycin D, cyclophosphamide, 6 azauridine, vincristine, hydroxyurea, 6 mercaptopurine and adriamycin. The fact that 47% of all women analysed in this series became pregnant and that only 5 women who wanted to have so far failed suggests that, after the cytotoxic chemotherapy regimens used in this series for treating gestational trophoblastic tumours, infertility is rarely a problem.

### IS *IN VITRO* DRUG SENSITIVITY DEPENDENT ON THE TYPE OF CLONOGENIC ASSAY?

P. J. HEPBURN, J. R. W. MASTERS AND B. T. HILL

*From the Institute of Urology & Laboratory of Cellular Chemotherapy, Imperial Cancer Research Fund, London*

CLONOGENIC assays are widely used to determine *in vitro* drug sensitivities. Particular interest has been generated by the recent work of Salmon and his colleagues showing that such assays for human tumours allow accurate prediction of clinical response in certain tumours (*Cloning of Human Tumour Stem Cells*, Ed. Salmon, 1980). There are many variants of these colony-forming assays, and the aim of this project was to compare several of them. We have used the human bladder-carcinoma cell line RT112 and determined its sensitivity to 2 drugs in common clinical usage, methotrexate and adriamycin.

Colony-forming ability was assessed directly on plastic, in the presence or absence of 3T3 feeder layers, using both exponentially growing cells and those in lag phase. Comparisons were also made between the colony formation of cells left *in situ* following drug treatment and those transferred and replated either immediately or 24 h after drug treatment.

Our results show that, depending on the assay procedure, the $ID_{50}$ (dose required to reduce survival by 50% in a 24h exposure) for MTX ranged from $5 \times 10^{-10}$M to $1 \times 10^{-7}$M, and for ADR from $7{\cdot}5 \times 10^{-9}$M to $3 \times 10^{-8}$M. We conclude that the clonogenic assay used is a significant variable in the measurement of *in vitro* drug sensitivity.

### SPIROGERMANIUM, A NEW TYPE OF ANTITUMOUR AGENT: CYTOTOXIC EFFECTS AND BIOLOGICAL ACTIVITY

R. D. H. WHELAN AND B. T. HILL

*From the Laboratory of Cellular Chemotherapy, Imperial Cancer Research Fund, Lincoln's Inn Fields, London WC2A 3PX*

THE lethal and biological effects of spirogermanium (2-aza-8-germanspiro 4,5 decane-2-propanamine, 8,8-diethyl-*N*,*N*-dimethyl dichloride) (NSC 192965) have been studied *in vitro*. Experiments using Syrian hamster ovary cells (NIL8) show that spirogermanium causes exponential kill with increasing dose, time and temperature. These lethal effects show no cell-cycle-phase specificity. Spirogermanium does not appear to interfere with cell cycling. Some preferential cytotoxicity was found when spirogermanium was tested against logarithmically growing rather than quiescent cells. Similar lethal effects occurred when spirogermanium was tested against cell lines derived from 2 human colonic carcinomas

(Lovo, COLO 205) and a human neuroblastoma (CHP 100) with $ID_{50}$ values of 0·52 to 0·74 μg/ml and $ID_{90}$ values of 1·2 to 1·8 μg/ml. Complete cell lysis occurred at concentrations of spirogermanium causing >2 log cell kill. Cell-membrane damage (as judged by the ability of COLO 205 cells to exclude trypan blue) resulting from spirogermanium treatment was both time- and dose-dependent. Spirogermanium does not alter cell volume, DNA, RNA or protein content, but it does inhibit $^3$H-leucine incorporation into protein at sublethal concentrations.

We propose a predominantly nonspecific mode of action for spirogermanium, affecting the cell membrane and protein synthesis, leading to cytolysis *in vitro*. This might explain the clinically observed side effect of local tissue damage after i.m. administration. Spirogermanium is currently undergoing Phase I/II clinical trials, and shows promise in previously treated ovarian tumours.

### ERYTHROCYTES AS INTRAVENOUS CARRIERS OF ANTI-NEOPLASTIC AGENTS

D. A. LEWIS AND C. M. RAYMONT

*From the Pharmocological Laboratories, Department of Pharmacy, University of Aston, Birmingham B4 7ET*

ENCAPSULATION of drugs in small vesicles, either synthetic or natural, has proved of limited use as a method of drug delivery, since such vesicles are rapidly removed by the reticuloendothelial system. However, drugs can be encapsulated in intact erythrocytes, which, when returned to the circulation, will survive for their normal lifespan (Jenner *et al.* (1981), *Br. J. Pharmacol.*, **73,** 212). Both cyclophosphamide (640 μg) and methotrexate (610 μg), encapsulated in 1 ml of packed red blood cells, survived up to 50 days when reinjected into the rat (the normal lifespan of the rat erythrocyte).

Using a methotrexate–fluorescein conjugate (F-MTX) (Gapski *et al.* (1975) *J. Med. Chem.*, **18,** 526) we showed that young erythrocytes will take up more drug than older ones. Such differential loading by selecting the age of the cell population may be of use clinically in maintaining effective blood levels of a drug without the large fluctuations often observed with oral administration.

Thus encapsulation of anti-neoplastic agents in erythrocytes may prove a useful delivery system for anti-cancer drugs (either singly or in combination regimes) especially as a "maintenance" therapy or for removing residual tumour cells after surgery.

### DEVELOPMENT OF RADIATION-RESISTANT CELL LINES *IN VITRO* AND A STUDY OF THEIR DRUG SENSITIVITIES

A. S. BELLAMY AND B. T. HILL

*From the Laboratory of Cellular Chemotherapy, Imperial Cancer Research Fund, London WC2 3PX*

CERTAIN human tumours recurring after radiotherapy have shown a reduced response to subsequent chemotherapy (Price *et al.* (1977), *Oncology*, **35,** 26). The aim of these studies is to determine whether exposure of tumour cells *in vitro* to radiation also leads to drug resistance.

Two cell lines were investigated: the murine L5178Y lymphoma and a human line (HN-1) derived from a primary squamous-cell carcinoma of the tongue (Easty *et al.* (1981) *Br. J. Cancer*, **43,** 772). The murine and human cell lines received 10 or 11 fractions of 2 Gy or 4·5 Gy per fraction respectively. This schedule for the human line attempted to mimic clinical radiotherapy.

These irradiated lines were compared with the parent lines in terms of their X-ray response, chromosome number, growth rates, macromolecular content and cell-cycle distributions. Preliminary results indicate that irradiation did not appear to change any of these parameters. However, more detailed analyses of radiosensitivity and chromosome karyotyping are under way.

Responses of the irradiated and parent cell lines to cytotoxic drugs are now being investigated; since no significant kinetic changes have been observed, any differences in sensitivity may represent the response of altered cell populations. Preliminary data suggest a change in response to the cytotoxic effects of bleomycin and *cis*-platinum after X-rays; other drugs being investigated are adriamycin, 5-fluorouracil, dibromodulcitol and hydroxyurea.

### THE STABILITY OF N-HYDROXYMETHYL COMPOUNDS DERIVED FROM N-METHYL CONTAINING ANTITUMOUR AGENTS

D. ROSS, A. GESCHER, J. A. HICKMAN AND M. F. G. STEVENS

*From the CRC Experimental Chemotherapy Group, Department of Pharmacy, University of Aston, Birmingham B4 7ET*

OXIDATIVE N-demethylation is thought to proceed *via* transient N-hydroxymethyl intermediates, which have been considered to break down to the N-desmethyl derivative and formaldehyde. These intermediates have been implicated in the antitumour effect of both hexamethylmelamine and aryldimethyltriazenes (Hickman (1978) *Biochimie*, **60**, 997).

We consider that N-hydroxymethyl intermediates have different stabilities which can be shown by a colorimetric assay for formaldehyde (Nash (1953) *Biochem. J.*, **131**, 555). Hydroxymethylpentamethylmelamine (HMPMM) will react directly with the formaldehyde-detecting Nash reagent, whereas a possible intermediate derived from the antitumour agent N-methylformamide (NMF), N-hydroxymethylformamide (HMF), gave a positive result only after the addition of 1*N* NaOH. As the urine of male CBA mice which had been treated with 400 mg/kg NMF i.p. only yielded a Nash-positive species under comparable alkaline conditions, the presence of HMF as a urinary metabolite of NMF is strongly indicated.

N-hydroxymethyl derivatives seem to have varying stabilities; there are those, such as the ones derived from aminopyrines, which decompose instantaneously to formaldehyde and the N-desmethyl compound; those, of an intermediate stability such as HMPMM, which react directly with Nash reagent; and those, typified by HMF, which are insensitive to Nash reagent in the absence of added alkali.

### THE ROLE OF ISOCYANATE FRAGMENTS IN THE ANTITUMOUR ACTIVITY OF CHLOROETHYLNITROSOUREAS

N. W. GIBSON AND J. A. HICKMAN

*From the CRC Experimental Chemotherapy Group, Department of Pharmacy, University of Aston, Birmingham B4 7ET*

THE importance of the isocyanate fragment in the *in vitro* cytotoxicity of chloroethylnitrosoureas (CENU) has not been yet resolved. We have investigated the cytotoxicity of CENU and their respective isocyanates (chloroethylisocyanate from BCNU, cyclohexylisocyanate from CCNU) in 2 cell lines which are sensitive to CENU *in vivo*

(TLX5 lymphoma, L1210 leukaemia) and to 2 which are resistant *in vivo* (TLX5R lymphoma, L1210R leukaemia). The TLX5 lymphoma is resistant *in vivo* to alkylating agents, whereas the L1210 leukaemia is highly sensitive.

The results (Table) indicate that the role of isocyanates in the cytotoxicity of CENU depends upon the cell line under investigation, since a comparison of the concentration required to give a 3 log cell kill against sensitive or resistant lines allows estimation of the selectivity of a cytotoxic fragment.

TABLE.—In vitro *sensitivity of cell lines to CENU and derived isocyanates, expressed as* $\mu$M *ti give a* 99·9% *cell kill.*

| Compound | TLX5 | TLX5R | L1210 | L1210R |
|---|---|---|---|---|
| BCNU | 25 | 96 | 17 | 60 |
| Chloroethyliso-cyanate | 53 | 98 | 60 | 92 |
| CCNU | 56 | 109 | 16 | 88 |
| Cyclohexyliso-cyanate | 138 | 256 | 234 | 208 |

We conclude that isocyanates may be important in the selective toxicity of CENU to the TLX5 lymphoma, but less so to the L1210 leukaemia.

### EFFECTS OF TREATMENT ON *IN VITRO* RESPONSES IN PATIENTS WITH MALIGNANT LYMPHOMA

M. D. WHITHAM AND B. W. HANCOCK

*From the Department of Medicine, Royal Hallamshire Hospital, Sheffield*

IN THIS study one aspect of cellular immunity, lymphocyte transformation induced *in vitro* by the mitogens phytohaemagglutinin (PHA), concanavalin A (Con A) and pokeweed mitogen (PWM) has been used to assess 8 patients with Hodgkin's disease (HD) and 4 patients with non-Hodgkin's lymphoma (NHL) as both the disease state and the treatment have been shown to affect immunity in such patients (Hancock *et al.* (1977) *Clin. Oncol.*, **3**, 137).

Seven of the HD patients received cyclical chemotherapy, and 1 received radiotherapy. In the chemotherapy group those with initial normal responses (4 PHA, 2 Con A, 5 PWM) showed a drop to subnormal within the first 3 cycles; these, together with the initially subnormal patients, have remained subnormal to date (minimum 4 months, maximum 13 months). The radiotherapy patient had initially normal PHA and PWM responses, which fell to subnormal on irradiation.

Three of the NHL patients received radiotherapy and one received chemotherapy. The radiotherapy patients all had initially normal Con A responses, 2 had normal PHA and PWM responses. After treatment, all had subnormal responses which gradually returned to near pretreatment levels. The chemotherapy patient had an initially normal PHA response which fell on treatment, but has (together with Con A and PWM responses) returned to normal within 6 months.

This continuing study has already shown that lymphocyte transformation can be used as one index of cellular immunity in patients with malignant lymphoma undergoing treatment. Chemotherapy has a more prolonged depressive effect on immunity than has radiotherapy.

### QUANTITATIVE HISTOLOGICAL STUDIES OF VOLUMETRIC CHANGES IN EPIDERMAL APPENDAGES DURING DMBA-CARCINOGENESIS OF HAMSTER SKIN

B. AL-AZZAWI AND F. H. WHITE

*From the Department of Human Biology and Anatomy, University of Sheffield, Sheffield S10 2TN*

MOST morphological studies on cancer development have been essentially subjective, and methods which enable the generation of more objective information from histological sections might provide more valuable information on human and experimental cancer and precancer. The advent of automated analysing devices, coupled with the powerful biological tool of stereology, has now made the acquisition of such information possible. The present report deals with the morphometric aspects of epidermal appendages during experimental carcinogenesis. In particular we are concerned with evaluating changes in epidermal appendages, which have been inadequately documented in experimental studies. A 0·5% solution of DMBA in liquid paraffin was applied to the shaved dorsal skin of male Syrian golden hamsters for up to 7 weeks. Five untreated animals served as controls and tissue samples from 5 animals were obtained 9, 12 and 16 weeks after the start of DMBA application. Using an MOP (Kontron) semi-automatic image-analysing system, the volume densities of hair follicles ($V_{HF}$) and sebaceous glands ($V_{SG}$) were determined in both the dermis and hypodermis for the normal and carcinogen-treated groups. In the dermis, $V_{HF}$ showed little change from normal during carcinogenesis, but $V_{SG}$ increased almost 3-fold. In the hypodermis, both $V_{HF}$ and $V_{SG}$ decreased markedly compared with normal skin. The differential changes in appendages in the connective-tissue components cannot as yet be explained, but the methods used have generated reliable objective data which can be obtained from any routine histological material. Wide application of these techniques might provide valuable insights into the pathogenesis of malignancy.

### VARIATIONS IN COLLAGEN FIBRIL DIAMETER IN THE LAMINA PROPRIA DURING HAMSTER CHEEK-POUCH CARCINOGENESIS

S. G. TARPEY, N. J. SMITH AND F. H. WHITE

*From the Department of Human Biology and Anatomy, University of Sheffield, Sheffield S10 2TN*

THE development of squamous-cell carcinomas is often accompanied by changes in the underlying connective tissue which include both the infiltration of inflammatory cells and the apparent destruction of collagen. In the present report we investigate the dimensions of collagen fibrils with a view to determining the nature of any dimensional alterations during defined stages of cancer development in an experimental *in vivo* system. A 0·5% solution of DMBA in liquid paraffin was applied to cheek pouches of 15 male Syrian golden hamsters 3 × /week for up to 15 weeks. Samples of mucosa were processed for electron microscopy and 1 μm sections with toluidine blue were used to assign 5 blocks from each of 5 animals to hyperplasia, dysplasia and carcinoma groups. Five untreated animals were used as controls. By the use of a stratified sampling scheme, electron micrographs of lamina propria at a final magnification of 90,000 were recorded from regions adjacent to the epithelial cells at each of the stages. Measurements of transversely sectioned collagen fibrils were made using an MOP (Kontron) image analyser. Routine ultrastructural examination revealed progressive disorganization of collagen fibres in the lamina propria, accompanied by fibroblast changes. These included increased width of rough endoplasmic reticulum cisternae. The quantitative data described a progressive reduction in collagen-fibril diameter during neoplastic development. Our observations suggest that significant changes in collagen synthesis are occurring in the lamina propria during experimental carcinogenesis. Further work is necessary to elucidate the role of these changes in the development of malignancy and premalignancy.

### RETROVIRUS PARTICLES ASSOCIATED WITH CANINE LYMPHOSARCOMA AND LEUKAEMIA

F. M. TOMLEY*, S. J. ARMSTRONG*, P. A. NUNES DE SOUZA†, T. G. WREGHITT‡, L. N. OWEN* AND B. W. J. MAHY†

*From the *Oncology Unit, Department of Clinical Veterinary Medicine, †Virology Division, Department of Pathology, ‡Public Health Laboratory Service, Cambridge*

A STUDY of short-term cultures of canine lymphosarcomatous and leukaemic tissue indicates the presence of virus particles which have properties similar to those of known retroviruses.

Cells from 58 spontaneous cases of canine lymphosarcoma and leukaemia have been examined. 64% were positive for poly(rC)-oligo(dG)-templated reverse transcriptase (RT) activity, compared to 13% of 30 normal dogs. This RT activity was found to band in sucrose at 1·15–1·18 g/ml. $^3$H-uridine-labelled culture supernatants were found to contain particles which banded in this same density range in 83% of the cases, compared to 9% of normal dogs.

Preparations of negatively stained material from RT-positive cultures were examined by electron microscopy, and numerous particles resembling the cores of known retroviruses were seen. Particles were of total diameter ~65 nm and contained a nucleoid of diameter ~35 nm surrounded by a membrane bearing an ill-defined fringe with projections 9 nm long. Particles of 100–140 nm have been seen only rarely.

Further biochemical examination of particles from 3 cases showed that they appear to contain a high-mol.-wt RNA (60–70S) as shown by glycerol gradient rate-zonal centrifugation. Simultaneous detection assays (Schlom & Spiegelman (1971) *Science*, **174,** 840) indicated that this RNA is associated with RT activity as RNA-[$^3$H]cDNA complexes could be detected at both 35S and 60–70S in glycerol.

We conclude that we have detected a retrovirus in dogs which is present in the normal population but is also found associated with most lymphosarcoma and leukaemia cases. We have no evidence of an aetiological involvement of this virus in the disease process.

### ULTRASTRUCTURAL DISCRIMINATION BETWEEN TUMOURS OF EPITHELIAL AND FIBROBLASTIC ORIGIN: CAN A SPINDLE-CELL TUMOUR BE A CARCINOMA?

N. WILLMOTT*, P. G. TONER†, A. L. McLAY† AND K. C. CALMAN*

*From the *Department of Oncology, University of Glasgow, and the †Department of Pathology, Royal Infirmary, Glasgow*

IT IS WELL recognized that predicted tumour behaviour is a factor involved in determining the treatment of human malignancy. Such predictions may be made on the basis of tissue origin, or histogenesis, of the tumour, since this is often related to biological behaviour. The determination of histogenesis may be difficult, especially in the case of a tumour characterized on light microscopy by a spindle-cell pattern. This situation can be mimicked to some extent by transplantable animal tumours used as models of human disease, and if the experimental systems are to be fully understood it is important that they be characterized as precisely as current technology permits. Consequently, in this study we have examined ultrastructural features of a group of transplantable animal tumours in an attempt to correlate cellular differentiation with presumptive histogenesis. The tumours studied included a well differentiated adenocarcinoma and poorly differentiated spindle-cell sarcomas, which we took as 2 extremes of malignant epithelial-cell differentiation. These tumours were compared with a transplantable rat tumour (Sp15) which although of predominantly spindle-cell pattern had arisen in the mammary region of a female rat and metastasized principally to regional lymph nodes. This pattern of behaviour is most compatible with an epithelial origin. Moreover, when ultrastructural features (cellular adhesion specializations, basal lamina production, and cytoplasmic differentiation) of the tumour were examined, there were vestiges of epithelial differentiation. These observations are consistent with the proposition that the designation "spindle-cell tumour" does not necessarily exclude certain tumours of epithelial origin.